# Recent Advances in Polymer Science and Fabrication Processes for Enhanced Microfluidic Applications: An Overview

**DOI:** 10.3390/mi15091137

**Published:** 2024-09-06

**Authors:** María F. Alexandre-Franco, Rahmani Kouider, Raúl Kassir Al-Karany, Eduardo M. Cuerda-Correa, Awf Al-Kassir

**Affiliations:** 1 Departamento de Química Orgánica e Inorgánica, Facultad de Ciencias, Universidad de Extremadura, Avenida de Elvas s/n, 06006 Badajoz, Spain; malexandre@unex.es; 2 Department of Technology, Ziane Achour University of Djelfa, Djelfa 17000, Algeria; k.rahmani@univ-djelfa.dz; 3 School of Industrial Engineers, University of Extremadura, 06006 Badajoz, Spain; ralxkass@alumnos.unex.es (R.K.A.-K.); aawf@unex.es (A.A.-K.)

**Keywords:** microfluidic applications, polymer fabrication, biocompatible polymers, advanced microfluidics, organ-on-chip technology

## Abstract

This review explores significant advancements in polymer science and fabrication processes that have enhanced the performance and broadened the application scope of microfluidic devices. Microfluidics, essential in biotechnology, medicine, and chemical engineering, relies on precise fluid manipulation in micrometer-sized channels. Recent innovations in polymer materials, such as flexible, biocompatible, and structurally robust polymers, have been pivotal in developing advanced microfluidic systems. Techniques like replica molding, microcontact printing, solvent-assisted molding, injection molding, and 3D printing are examined, highlighting their advantages and recent developments. Additionally, the review discusses the diverse applications of polymer-based microfluidic devices in biomedical diagnostics, drug delivery, organ-on-chip models, environmental monitoring, and industrial processes. This paper also addresses future challenges, including enhancing chemical resistance, achieving multifunctionality, ensuring biocompatibility, and scaling up production. By overcoming these challenges, the potential for widespread adoption and impactful use of polymer-based microfluidic technologies can be realized.

## 1. Introduction

Microfluidics, the manipulation of fluids within micrometer-sized channels, has become a cornerstone technology across various scientific disciplines, including biotechnology, medicine, and chemical engineering [1,2,3,4,5,6,7]. This field has revolutionized how complex problems are approached and solved by providing precise control over small volumes of fluids. The applications of microfluidics are vast, ranging from simple fluidic manipulation to highly sophisticated systems capable of performing complex biochemical assays [8,9], diagnostics [10], and even organ-on-chip models that replicate human organ functions on a microscale [11]. Microfluidics also plays a crucial role in directional liquid transport, enabling the precise manipulation of fluids at the microscale [12]. This technology leverages the control of surface tension and channel design to guide liquid movement in specific directions, which is essential for applications like self-cleaning surfaces, microfluidic circuits, and targeted drug delivery systems. Despite significant advancements in designing open surfaces for liquid manipulation, achieving diode-like liquid transport within enclosed spaces remains a challenging task [13]. By creating channels with tailored wettability and geometric features, microfluidics facilitates the efficient and controlled transport of liquids, enhancing the functionality of advanced materials and devices. Hence, microfluidics has drawn the attention of the scientific community in the last years. 

**Figure 1:**
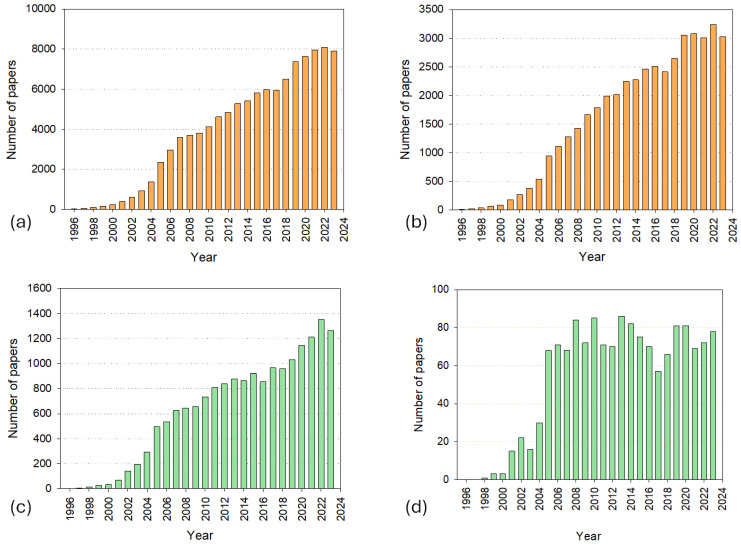
Number of papers published in the period 1997–2023 with the term “microfluidic(s)” (**a**) in the title, abstract, and/or keywords; (**b**) only in the title. Number of papers published in the period 1997–2023 with the terms “microfluidic(s)” and “polymer(s)” (**c**) in the title, abstract, and/or keywords; (**d**) only in the title. Source: Scopus.

Figure 1 illustrates the publication trends in the field of microfluidics, with a specific focus on polymer-based microfluidic devices over the period from 1997 to 2023. Figure 1a,b show the number of publications that include the terms “microfluidic” or “microfluidics” in the title, abstract, and/or keywords, with Figure 1a covering all relevant terms and Figure 1b focusing solely on those terms in the title. Similarly, Figure 1c,d focus on publications that also include “polymer” or “polymers” in conjunction with the terms “microfluidic” or “microfluidics”.

From Figure 1, it becomes apparent that in the early years, the data clearly show an exponential growth in the number of publications, particularly until around 2006. This initial surge can be attributed to the rapid advancements and the burgeoning interest in microfluidic technologies during the early 2000s, a period marked by significant foundational developments. Researchers were actively exploring the potential of microfluidics across various fields, leading to a steep increase in related publications as new techniques and applications were being established. However, after 2006, the growth trend becomes more linear rather than exponential. This shift likely reflects the maturation of the field. As foundational knowledge and technologies became more established, the rate of groundbreaking discoveries decreased, leading to a more steady, linear increase in publications. During this period, the research focus may have shifted towards refining existing technologies, exploring specialized subdomains within microfluidics, and applying microfluidic principles to new areas of research.

Figure 1d highlights an interesting trend: the number of publications specifically ad-dressing the combination of microfluidics and polymers has remained relatively constant after 2006. This stabilization might be due to several factors. First, as the field matured, there was likely a shift from exploratory, high-impact studies to more application-oriented research, which may not always result in the same volume of publications. Second, the diversification of research into niche areas within microfluidics could mean that while the overall field continues to grow, the number of publications explicitly focusing on both microfluidics and polymers does not exhibit the same growth, especially if such research is categorized under more specialized topics. Furthermore, the growth of proprietary research and development in industry settings could play a role in this trend. As microfluidic technologies transitioned from academic research to industrial applications, there may have been an increase in unpublished proprietary studies, particularly those related to product development and commercialization.

The evolution of microfluidics from basic fluidic manipulation to such advanced systems has been facilitated by significant advancements in materials science, particularly the utilization of polymers [14,15,16]. Polymers, both natural and synthetic, offer numerous advantages that make them indispensable in the fabrication of microfluidic devices. These advantages include flexibility [17], biocompatibility [18], and structural integrity [19], which are essential for creating devices that can withstand various mechanical and chemical stresses while maintaining their functionality. Moreover, polymers can be easily processed and modified, making them ideal for developing innovative microfluidic technologies [16,20,21,22]. Hence, polymers have been used in the fabrication of microfluidic devices for several decades. This can be easily appreciated from Figure 1c,d, which illustrate the total number of published papers with the terms “microfluidic”/“microfluidics” AND “polymer”/”polymers” in the title, abstract, and/or keywords or only in the title, respectively. In the former case, the above-mentioned exponential rise can also be observed, with over 1200 papers published yearly in the last four years (2020–2023). Nonetheless, the number of papers that include both search terms only in the title remains almost constant around 80 papers per year, which is worth noting.

One of the critical aspects of polymer use in microfluidics is their ability to provide essential support in creating precisely controlled microenvironments necessary for fluid and particle manipulation [23]. This precise control is crucial for applications such as cell culture, drug delivery, and chemical synthesis, where the environment’s micro-scale features significantly impact the outcomes. Polymers’ diverse physical and chemical properties, such as mechanical strengths, chemical resistances, optical properties, and electrical conductivities, can be tailored to meet specific application requirements, enhancing the versatility and applicability of microfluidic devices [21].

The versatility of polymers in microfluidic applications is attributed to their diverse range of physical and chemical properties. For instance, polymers can be designed to be flexible or rigid, transparent or opaque, conductive or insulating, depending on the needs of the application. This adaptability makes polymers suitable for a wide range of microfluidic devices, from diagnostic tools to lab-on-a-chip systems.

Moreover, the development of new polymeric materials and advanced fabrication techniques has significantly enhanced the performance and broadened the application scope of microfluidic devices [24,25]. Innovations in polymer science have led to the creation of materials with improved mechanical properties, enhanced optical clarity, superior chemical resistance, and better biocompatibility. These advancements have not only improved the functionality and reliability of microfluidic devices but have also opened up new possibilities for their use in various fields, including healthcare, environmental monitoring, and industrial processes.

Microfluidic devices play a pivotal role in biomedical applications, providing platforms for high-throughput screening [26], disease diagnostics [10], and personalized medicine [27]. For example, microfluidic chips can be used to perform rapid blood tests [28], analyze DNA sequences [29], or culture cells in controlled environments that mimic the human body [30,31]. In environmental science, microfluidics enables the detection of pollutants and pathogens in water and air [32,33,34], offering real-time monitoring and rapid response capabilities [35,36,37]. In industrial processes, microfluidic systems facilitate precise chemical reactions [38,39] and material synthesis [40,41], enhancing efficiency and reducing waste.

Advancements in microfluidic technology have also spurred the development of organ-on-chip models, which simulate the physiological functions of human organs [42,43,44,45]. These models provide valuable insights into disease mechanisms and drug interactions, potentially reducing the need for animal testing and accelerating the development of new therapies. Furthermore, the integration of microfluidics with other technologies, such as sensors and actuators, has led to the creation of smart microfluidic systems that can perform complex tasks autonomously [46,47]. Unlike traditional closed microchannel microfluidic systems, free-boundary microfluidic manufacturing (FBMM) processes continuous precursor fluids into jets or droplets within a more open environment. FBMM is particularly valued for its exceptional flexibility, stability, cost-effectiveness, user-friendliness, and versatility in producing advanced materials and structures [48].

Despite these advancements, several challenges remain in the field of microfluidics, particularly related to the materials used in device fabrication. The selection of suitable polymers that can withstand the operational conditions of microfluidic devices, such as chemical exposure and mechanical stress, is critical [49,50]. Additionally, the fabrication processes must ensure the precision and reproducibility of microfluidic features to maintain device performance.

This review aims to explore the significant advancements in polymer science and fabrication processes that have enhanced the performance and broadened the application scope of microfluidic devices. Various fabrication techniques, such as replica molding, microcontact printing, solvent-assisted molding, injection molding, and 3D printing, will be examined, highlighting their advantages, limitations, and recent developments. Additionally, advancements in polymer materials, including flexible and wearable microfluidic devices, improved mechanical properties, enhanced optical properties, superior chemical resistance, and electrical conductivity, will be discussed.

Furthermore, advanced fabrication techniques such as nanoimprint lithography, laser micromachining, hybrid fabrication techniques, focused ion beam (FIB) micromachining, and electrochemical micromachining will be explored. These techniques have enabled the creation of more complex and multifunctional microfluidic devices, pushing the boundaries of what can be achieved in this field.

In addition to fabrication techniques and materials development, the diverse applications of polymer-based microfluidic devices in biomedical applications, environmental monitoring, industrial processes, point-of-care diagnostics, organ-on-chip models, and personalized medicine will be explored. Each section will provide an in-depth analysis of how these devices are being used to address current challenges and improve outcomes in these fields.

**Figure 2:**
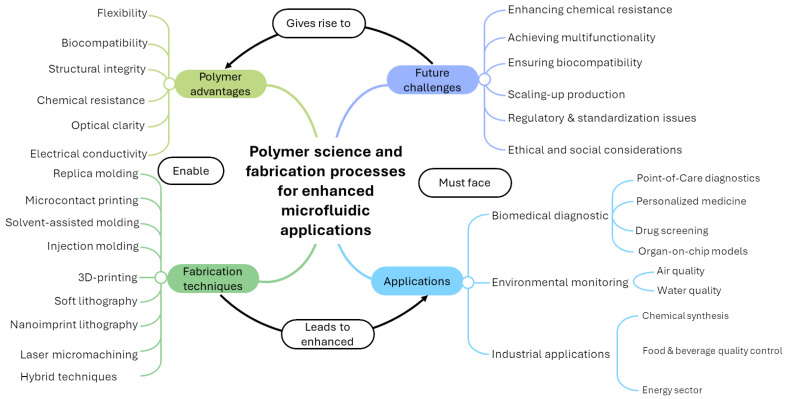
A schematic representation of the main aspects of the role of polymer science in the fabrication processes for enhanced microfluidic applications.

Finally, the future challenges and developments in polymer-based microfluidic devices will be addressed, including chemical resistance and gas permeability, integration of multifunctional systems, biocompatibility and biofouling, scalability and manufacturing, regulatory and standardization challenges, and ethical and social considerations. By addressing these challenges, the path for the widespread adoption and impactful use of these transformative technologies will be paved. A schematic representation of the topics dealt with in this review, as well as of the relations that can be established between them, is shown in Figure 2.

## 2. Fabrication Techniques

The fabrication of polymer-based microfluidic devices involves several sophisticated techniques, each contributing uniquely to the precision and efficiency of the resultant microstructures. Some key methods are detailed in the following sections.

### 2.1. Photolithography

**Figure 3:**
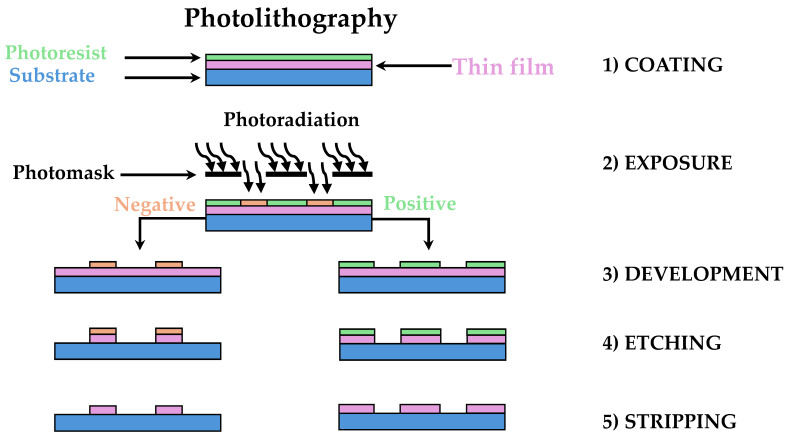
Schematic representation of the photolithographic process.

Photolithography is one of the most established and widely used techniques in the microfabrication of microfluidic devices [51]. It involves the use of light to transfer geometric patterns from a photomask onto a photosensitive material, typically a photoresist, which is coated on a substrate. This technique is integral to the creation of high-precision microstructures and has been extensively employed in both academic research and industrial applications of microfluidics. Figure 3 illustrates the photolithographic process. A photoresist solution is typically applied to a flat substrate, which is then spun into a thin film and dried. When this photosensitive layer is exposed to UV light through a photomask, a transparent plate with the desired pattern in an opaque metallic film, the regions of the photoresist that are exposed to light undergo chemical changes. The type of photoresist determines these changes. In a positive photoresist, exposure to light breaks down the polymer molecules, increasing their solubility in a specific developer solution compared to the unexposed areas. In contrast, a negative photoresist undergoes photochemical crosslinking upon exposure to light, making the exposed regions nearly insoluble in the developer. After exposure, the process continues with etching and stripping steps, resulting in the final structure.

Photolithography is renowned for its ability to produce microstructures with extremely high resolution and precision, often down to the sub-micron scale [52]. This level of accuracy is crucial for the fabrication of complex microfluidic devices where the exact dimensions of channels and features directly influence fluid dynamics and device performance. The technique is particularly well-suited for creating intricate and dense networks of microchannels, which are essential for applications such as lab-on-a-chip systems, microreactors, and bioassays [53]. One of the key advantages of photolithography is its scalability and repeatability [54]. The process is highly standardized and can be scaled up for mass production with consistent quality. This makes it ideal for commercial applications where large quantities of identical microfluidic devices are required. The use of photomasks allows for the rapid replication of microstructures across multiple substrates, enabling high-throughput manufacturing [55]. Photolithography is also highly versatile, as it can be used with a wide range of substrates, including silicon, glass, and various polymers [56]. This versatility allows for the fabrication of microfluidic devices with different physical, chemical, and optical properties, tailored to specific applications. Moreover, photolithography can be integrated with other microfabrication techniques, such as etching and deposition, to create more complex and multifunctional devices [57].

Despite its advantages, photolithography has several limitations, particularly when applied to the fabrication of microfluidic devices. One of the main drawbacks is the high cost and complexity of the process. Photolithography requires expensive equipment, such as mask aligners and cleanroom facilities, which can be prohibitive. The preparation of photomasks, especially for custom or intricate designs, can also be time-consuming and costly. Another limitation is the inherent restriction in the range of materials that can be processed using photolithography. The technique is primarily suited for rigid and flat substrates, making it less compatible with flexible or curved surfaces [58]. Additionally, the process typically involves multiple steps, including coating, exposure, development, and etching, each of which must be precisely controlled to achieve the desired results. Any deviation in these steps can lead to defects such as undercutting, over-etching, or misalignment, which can significantly impact the performance of the microfluidic device. The environmental impact of photolithography is another concern, as it involves the use of hazardous chemicals in the development and etching processes [59]. The disposal of these chemicals requires stringent environmental controls, adding to the operational costs and complexity of the process.

Recent advances in photolithography have focused on overcoming its limitations and expanding its applications in microfluidic device fabrication. One significant development is the introduction of advanced photoresists that offer improved sensitivity and resolution. These new materials allow for finer feature sizes and more complex patterns to be created, thereby enhancing the capability of photolithography in microfluidics. Another important advancement is the use of deep ultraviolet (DUV) and extreme ultraviolet (EUV) photolithography, which enables the fabrication of even smaller features with higher aspect ratios [60]. These techniques have been instrumental in pushing the boundaries of microfluidic device miniaturization, allowing for the creation of highly integrated systems with multiple functionalities on a single chip. The development of novel photomasks and maskless lithography techniques has also significantly improved the flexibility and cost-effectiveness of photolithography [61,62]. Maskless lithography, in particular, eliminates the need for expensive photomasks by directly writing the pattern onto the photoresist using a focused light source, such as a laser or electron beam. This approach is especially useful for rapid prototyping and for producing small batches of custom-designed microfluidic devices. Additionally, advances in soft lithography, a technique that often uses photolithographically created masters, have enabled the combination of photolithography with other fabrication methods to produce hybrid microfluidic devices. These developments have broadened the range of materials and structures that can be fabricated, leading to more versatile and application-specific microfluidic systems [63].

Despite the significant progress made in photolithography, several challenges remain in its application to microfluidic device fabrication. One of the key challenges is further reducing the cost and complexity of the process to make it more accessible for a wider range of applications [64,65]. While advances in maskless lithography and other techniques have addressed some of these issues, the need for cleanroom facilities and specialized equipment continues to be a barrier for many potential users [66]. Another challenge is improving the compatibility of photolithography with non-traditional substrates and materials. The development of new photoresists and processing techniques that can be applied to flexible, stretchable, or biocompatible materials would significantly expand the potential applications of photolithography in microfluidics, particularly in the areas of wearable technology and biomedical devices [67,68]. Furthermore, as the demand for more complex and multifunctional microfluidic devices grows, there is a need for continued innovation in photolithography to enable the fabrication of three-dimensional microstructures and multi-layered devices. Techniques such as multi-photon lithography, which allows for 3D patterning within a single step, are promising in this regard [69,70], but further research and development are required to make these techniques more practical and widely applicable.

### 2.2. Soft Lithography

Soft lithography is a set of techniques that use elastomeric stamps, molds, and conformable photomasks to create patterns on substrates. This method is widely used for fabricating microfluidic devices due to its versatility, simplicity, and ability to produce high-resolution features. Recent advances in soft lithography include the development of new elastomeric materials with improved mechanical and chemical properties. For example, researchers have developed UV-curable elastomers that offer higher resolution and faster processing times compared to traditional PDMS. UV curable elastomers also offer flexibility in alignment and bonding during the fabrication process, allowing for better chemical resistance, permeability, and flexible surface treatments compared to other known polymeric materials [71]. These elastomers can be applied between two surfaces and left without bonding until exposed to UV light, providing greater control during the fabrication process [72]. Also, they enable the fabrication of microfluidic chips with detailed features, such as microchannels as small as 100 μm wide and 3 μm deep, using UV lithography and molding processes [73]. The use of UV curable elastomers allows for rapid prototyping of microfluidic devices, with the ability to create micropillars, deep channel microfluidic structures, and multi-layer devices using UV-curing processes. The bonding strengths of UV curable elastomers are comparable to traditional materials like PDMS, making them suitable for various bonding techniques in microfluidic applications. Additionally, the integration of soft lithography with other microfabrication techniques, such as 3D printing and nanoimprint lithography, is expanding the capabilities of this method. The potential for enhanced optical quality, improved handling, and processing efficiency, characterized by shorter curing times and simplified procedures using UV light compared to traditional PDMS is also a key potentiality of UV curable elastomers [74]. Highly fluorinated UV curable polymers have been designed for microfluidic and combined microfluidics and optics applications [75]. 

While UV curable elastomers offer advantages, they may face challenges related to distortion and resolution problems, possibly due to higher-than-expected viscosity, particularly in the replication of smaller features [76]. The use of UV curable elastomers may require careful consideration of bonding techniques and material properties to ensure optimal performance in mechanically demanding applications [77].

Analogously, soft lithography, while widely used, has limitations such as time-consuming and expensive fabrication processes, which can limit platform complexity and hinder rapid device iterations [78,79]. Hybrid approaches combining conventional photolithography and two-photon polymerization have been introduced to fabricate master molds for soft lithography, offering convenience and complex 3D structures with high resolution [80].

Soft lithography techniques include replica molding, microcontact printing (μCP), micromolding in capillaries (MIMIC), and microtransfer molding. All these techniques are exposed in the following paragraphs.

#### 2.2.1. Replica Molding

**Figure 4:**
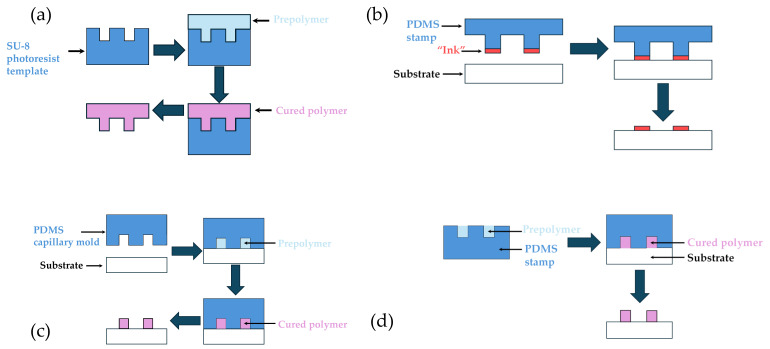
The four fundamental methods of soft lithography: (**a**) replica molding; (**b**) microcontact printing; (**c**) micromolding in capillaries; (**d**) microtransfer molding.

Replica molding is one of the most widely used soft lithography techniques in the fabrication of microfluidic devices. It constitutes an attractive alternative to traditional methods such as photolithography, that can be costly and require cleanroom facilities [81]. It involves creating a negative mold from a master template, usually made of materials such as silicon or SU-8 photoresist, through techniques like photolithography. This mold is then used to cast a polymer, typically polydimethylsiloxane (PDMS), which solidifies to form a precise replica of the microstructures. A schematic representation of the process is illustrated in Figure 4a. PDMS is frequently used due to its desirable properties such as its flexibility and excellent resolution [82]. It also exhibits excellent biocompatibility, optical transparency, and ease of use, making it a staple material in microfluidic device fabrication. However, its application is not without limitations. One significant drawback of PDMS is its susceptibility to swelling when exposed to certain organic solvents. This swelling can lead to distortion of microchannels and can compromise the structural integrity of the device. Furthermore, PDMS is highly permeable to gases, which can be both an advantage and a disadvantage depending on the application. For instance, while gas permeability is beneficial for cell culture applications, where gas exchange is necessary, it poses a challenge for applications requiring precise control over volatile reagent concentrations. Addressing these limitations requires the development of surface modifications or alternative materials that retain the advantageous properties of PDMS while mitigating its drawbacks.

Replica molding allows for rapid and cost-effective production of microfluidic devices with high fidelity to the master design [83]. Notably, PDMS’s transparency and biocompatibility make it ideal for biological and optical applications, enabling real-time observation of fluidic processes [84]. The flexibility, simplicity, and cost-effectiveness of replica molding make it an attractive method for producing microfluidic devices. 

One of the primary advantages of replica molding is its ability to produce microfluidic devices with high resolution and fidelity [85,86]. The technique can replicate features down to the nanoscale, making it suitable for creating complex microfluidic channels and networks that require precise geometrical accuracy. The high resolution is particularly important in applications such as lab-on-a-chip systems, where small variations in channel dimensions can significantly impact fluid flow and device performance. Replica molding is also highly versatile in terms of the materials that can be used [87,88]. Although PDMS is the most commonly employed material due to its biocompatibility, optical transparency, and flexibility, other polymers such as polyurethane and epoxy resins can also be used depending on the application requirements. This versatility allows for the customization of microfluidic devices with specific mechanical, chemical, and optical properties [89,90]. Another significant advantage is the low cost and accessibility of the technique [86,91]. Replica molding does not require expensive equipment or cleanroom facilities, making it feasible for a wide range of laboratories and industrial settings. The molds can be reused multiple times, further reducing the cost of fabrication. Additionally, the process is relatively straightforward and does not require extensive expertise, which facilitates rapid prototyping and iterative design of microfluidic devices.

Despite its advantages, replica molding has several limitations. One of the key challenges is the mechanical instability of PDMS, the most commonly used material in this technique. PDMS is prone to deformation under mechanical stress, which can lead to changes in channel dimensions and affect the performance of the microfluidic device. Additionally, PDMS has a tendency to absorb small hydrophobic molecules, which can interfere with certain biological and chemical assays, limiting its use in applications where chemical inertness is critical [92]. Another limitation is the potential for defects during the molding process. Air bubbles, incomplete filling, or imperfections in the master mold can result in defects in the final microstructures. These defects can compromise the functionality of the microfluidic device, particularly in applications requiring high precision. Furthermore, the manual steps involved in replica molding, such as mold alignment and polymer casting, can introduce variability and reduce the reproducibility of the devices, especially in high-throughput production scenarios. The scalability of replica molding is also a challenge. While the technique is ideal for small-scale production and prototyping, scaling up the process for mass production requires careful control over each step to maintain consistency and quality. The time-consuming nature of the mold preparation and curing processes further complicates efforts to scale up production.

Recent advances in replica molding have focused on addressing its limitations and expanding its capabilities for microfluidic applications. One significant development is the use of hybrid materials that combine the advantages of PDMS with other polymers to improve mechanical stability and chemical resistance [93,94]. For example, PDMS has been combined with polyimide to create microfluidic devices that are more resistant to mechanical deformation and less prone to absorption of hydrophobic molecules, making them suitable for a broader range of applications [95,96,97]. Another important advancement is the development of automated systems for the replica molding process. These systems can precisely control the polymer casting, curing, and demolding steps, reducing the risk of defects and increasing the reproducibility of the microfluidic devices. Automated systems also facilitate high-throughput production, making it easier to scale up the process for commercial applications. Additionally, the use of advanced surface treatments and coatings has improved the performance of replica-molded microfluidic devices [98]. For instance, surface modification of PDMS with fluorinated silanes has been shown to enhance its chemical resistance, allowing for its use in more chemically aggressive environments. Other treatments aim to reduce the surface roughness of the molded microstructures, thereby improving the optical clarity and flow characteristics of the microfluidic channels. Innovations such as multi-layer replica molding and integration with electronic components are also expanding the capabilities of PDMS-based microfluidic devices [99,100].

Despite these advancements, several challenges remain in the application of replica molding for microfluidic devices. One of the key challenges is further improving the mechanical and chemical properties of the materials used. While hybrid materials and surface treatments have provided some solutions, ongoing research is needed to develop polymers that offer the desired combination of flexibility, chemical resistance, and biocompatibility without compromising other essential properties. Another challenge is enhancing the scalability and automation of the replica molding process. Although automated systems have improved reproducibility and throughput, achieving consistent quality across large-scale production runs remains difficult. Developing faster curing methods and more efficient mold preparation techniques could help overcome these challenges and make replica molding more viable for mass production. Furthermore, there is a need to explore new materials that can be used with replica molding to expand the range of applications for microfluidic devices. For example, integrating conductive or optically active materials into the replica molding process could enable the fabrication of multifunctional devices with embedded sensors or optical components. This would open up new possibilities in areas such as environmental monitoring, point-of-care diagnostics, and wearable technology.

#### 2.2.2. Microcontact Printing (μCP)

Microcontact printing is a versatile technique where a patterned elastomeric stamp is used to transfer materials onto a substrate. This method excels in fabricating microarrays and functionalizing surfaces with biomolecules, facilitating the development of bioassays and diagnostic devices [99,101]. The process involves creating a stamp, often from PDMS, which is then inked with the desired material. The stamp is brought into contact with the substrate, transferring the inked pattern. The process is schematically illustrated in Figure 4b. This technique allows for the precise deposition of various materials, including proteins, DNA, and nanoparticles, enabling the creation of complex biochemical patterns necessary for advanced diagnostic applications [102,103].

Microcontact printing has been extensively used in creating biosensors, cell culture platforms, and tissue engineering scaffolds [104,105]. For instance, this technique can pattern extracellular matrix proteins on a substrate to guide cell adhesion and growth [106], which is crucial for developing tissue models and studying cell behavior. Microcontact printing is used for patterning surfaces with biomolecules and other materials, thus producing molecular micro- and nano-patterns of various biomolecules [107,108]. Recent innovations include the use of microcontact printing to fabricate flexible and stretchable electronics, which can be integrated with microfluidic devices for real-time monitoring and control [109,110]. It has found applications in biosensors, microarrays, and biomedical applications [103].

Despite its versatility, microcontact printing faces challenges such as achieving uniform material transfer and maintaining pattern fidelity over large areas [103,111,112]. Also, large-scale implementation of μCP has been challenging due to issues with reproducibility and uniform transfer of inked molecules over large areas. These issues are currently being addressed by developing new stamp materials with improved mechanical properties and exploring alternative printing techniques such as nanoimprint lithography [112,113,114]. Future directions include the integration of microcontact printing with other microfabrication methods to create multi-functional devices [115,116] and the development of high-throughput printing systems for large-scale production [113,117,118].

#### 2.2.3. Micromolding in Capillaries (MIMIC)

Micromolding in capillaries (MIMIC) is a versatile soft lithography technique widely used in the fabrication of microfluidic devices. As illustrated in Figure 4c, the method involves filling micro-scale capillaries, created by placing an elastomeric mold on a substrate, with a liquid pre-polymer. The polymer is then allowed to cure, resulting in the formation of microstructures that mirror the shape of the capillaries. Capillary microfluidic devices are self-powered, easy to use, and well established for diagnostics and drug monitoring, making them suitable for enhancing medication adherence [119]. Capillary microfluidic chips have been extensively studied and applied in various fields due to their unique advantages of low preparation difficulty, low material cost, excellent chemical properties, and flexible design [120]. This method offers a high level of control, making it a versatile and promising technique [121].

One of the key advantages of MIMIC is its capacity to produce microfluidic channels with high aspect ratios and complex geometries, which are difficult to achieve with other microfabrication techniques [122,123]. The ability to fabricate intricate and continuous microstructures makes MIMIC an ideal choice for creating microfluidic devices that require precise control over fluid flow, such as in lab-on-a-chip systems, microreactors, and cell culture platforms. MIMIC is also a low-cost and accessible technique as it does not require expensive equipment or cleanroom facilities [124,125,126]. The process is relatively simple and can be carried out in a standard laboratory setting, making it an attractive option for researchers and small-scale producers of microfluidic devices. Additionally, the elastomeric molds used in MIMIC are reusable, further reducing the overall cost of device fabrication. Another significant advantage is the versatility of the materials that can be used in MIMIC. The technique is compatible with a wide range of polymers and pre-polymers, allowing for the creation of microfluidic devices with tailored chemical, mechanical, and optical properties. This flexibility enables the design of devices for specific applications, such as those requiring chemical resistance, biocompatibility, or optical transparency.

Despite its advantages, MIMIC has certain limitations that can impact its effectiveness in microfluidic device fabrication. One of the primary challenges is the need for precise control over the capillary filling process. Incomplete filling, air bubble formation, or inconsistent flow of the pre-polymer can lead to defects in the final microstructures, compromising the functionality of the microfluidic device [127,128]. Another limitation is the reliance on the surface properties of the substrate and mold. The wetting properties of the pre-polymer must be carefully controlled to ensure proper capillary action, which can be challenging when using different materials or when creating complex geometries. Additionally, the curing process may lead to shrinkage or distortion of the microstructures, particularly when using certain polymers, which can affect the accuracy and performance of the device. MIMIC also requires careful alignment of the mold and substrate, especially in multi-layered device fabrication. Misalignment can result in defective channels or connections between layers, which can be difficult to correct without restarting the process. This manual aspect of MIMIC can introduce variability and reduce the reproducibility of the devices, particularly when scaling up production.

Recent advances in MIMIC have focused on improving the precision, efficiency, and material compatibility of the technique for microfluidic applications [129]. One notable development is the use of advanced surface treatments and coatings to enhance the wetting properties of the pre-polymer, ensuring consistent capillary action and complete filling of the mold. These treatments can also reduce the adhesion between the cured polymer and the mold, facilitating easier demolding and reducing the risk of defects [130,131,132,133]. Another significant advancement is the integration of automated systems to control the capillary filling and curing processes. Automated systems can precisely regulate the flow of the pre-polymer, monitor the filling process in real-time, and adjust parameters to optimize the quality of the microstructures. These innovations are particularly important for high-throughput fabrication and for applications requiring high levels of reproducibility. Material science has also played a crucial role in advancing MIMIC. The development of new polymers and pre-polymers with tailored properties, such as reduced shrinkage, improved mechanical strength, and enhanced chemical resistance, has expanded the range of applications for MIMIC-fabricated microfluidic devices. Additionally, the use of hybrid materials that combine the advantages of different polymers has allowed for the creation of microstructures with specific functionalities, such as embedded sensors or responsive surfaces.

While MIMIC has seen significant advancements, several challenges remain in its application to microfluidic devices. One of the key challenges is the scalability of the technique. While MIMIC is well-suited for small-scale production, scaling up the process for mass production while maintaining the precision and quality of the microstructures is challenging. Automated systems can mitigate some of these issues, but further research is needed to develop scalable solutions that preserve the advantages of MIMIC. Another challenge is the development of materials that are both compatible with MIMIC and suitable for the specific demands of microfluidic applications. While the technique is versatile in terms of material selection, not all polymers exhibit the desired properties for high-performance microfluidic devices, particularly in terms of biocompatibility, chemical resistance, and mechanical durability. Ongoing research into new materials and surface treatments is essential to overcome these limitations. Finally, the complexity of the microstructures that can be achieved with MIMIC is still limited by the capillary action and curing process. Creating multi-layered devices with intricate internal structures requires precise control over each step of the process, which can be difficult to achieve consistently. Innovations in mold design, material science, and process automation will be crucial in pushing the boundaries of what can be achieved with MIMIC in microfluidics.

#### 2.2.4. Microtransfer Molding (µTM)

Microtransfer molding (µTM) is an advanced soft lithography technique used in the fabrication of microfluidic devices. As illustrated in Figure 4d, it involves transferring a pre-polymer from a mold onto a substrate where it solidifies, typically through thermal or UV curing, to create micro-scale features. This technique is highly valued in microfluidics for its ability to produce complex, high-resolution patterns in a wide range of materials, including polymers and hydrogels.

One of the primary advantages of microtransfer molding is its ability to create intricate microstructures with high fidelity and resolution [134]. This precision is crucial in microfluidic devices, where accurate channel dimensions and surface patterns directly influence fluid behavior and device performance. Unlike other molding techniques, µTM allows for the fabrication of multi-layered structures, enabling the integration of various functionalities within a single microfluidic chip [135,136]. Additionally, microtransfer molding is compatible with a variety of materials, including those that are challenging to process using conventional methods. This flexibility makes it possible to fabricate devices with diverse chemical, mechanical, and optical properties tailored to specific applications. For instance, µTM can be used to create microfluidic devices with enhanced chemical resistance, improved optical transparency, or specific surface properties conducive to cell culture and bioassays [137,138]. Another significant advantage is the relatively low cost and simplicity of the process. µTM does not require expensive cleanroom facilities or complex equipment. The ability to reuse molds multiple times further reduces the overall cost of device fabrication.

Despite its advantages, microtransfer molding has certain limitations. One of the primary challenges is the potential for defects during the transfer process, such as incomplete filling of the mold or misalignment between layers [139]. These defects can compromise the functionality of the microfluidic device, particularly in applications requiring precise fluid control. The technique also involves several manual steps, such as aligning the mold with the substrate, which can introduce variability and reduce reproducibility. This can be particularly problematic when scaling up production or when high-throughput fabrication is required. Moreover, the choice of materials for both the mold and the substrate is critical. Some materials may adhere too strongly to the mold, making it difficult to achieve clean release without damaging the microstructures. Conversely, inadequate adhesion can lead to incomplete transfer, resulting in defective features.

Recent advances in microtransfer molding have focused on improving the accuracy, efficiency, and scalability of the technique for microfluidic applications [140]. One notable development is the use of advanced materials for molds, such as fluorinated elastomers, which offer excellent non-stick properties. These materials facilitate clean release from the substrate, reducing the risk of defects and improving the overall quality of the fabricated microstructures [141]. Another significant advancement is the integration of automated systems to enhance the reproducibility and throughput of µTM. Automated alignment systems and precision control mechanisms have been developed to minimize human error and ensure consistent quality across multiple batches of microfluidic devices. These innovations are particularly important for commercial applications, where large-scale production with minimal variability is essential [142]. The use of hybrid materials, combining the advantages of different polymers, has also been explored to enhance the performance of microfluidic devices fabricated using µTM. For example, combining soft, flexible polymers with more rigid materials can create devices that are both durable and adaptable to various fluidic environments [135,136,140].

Despite these advancements, several challenges remain in the application of microtransfer molding for microfluidic devices. One of the key challenges is scaling up the technique for mass production while maintaining the high resolution and accuracy required for microfluidics. As µTM often involves manual or semi-automated steps, fully automating the process without compromising quality is a significant hurdle. Another challenge is expanding the range of materials that can be effectively used with µTM. While the technique is versatile, not all materials exhibit the necessary properties for successful transfer molding, particularly when complex, multi-layered structures are required. Research is ongoing to develop new materials and surface treatments that can enhance the compatibility and performance of µTM in microfluidic applications. Additionally, the durability and long-term stability of microfluidic devices fabricated using µTM need to be further investigated, especially for applications involving harsh chemical environments or extreme mechanical stresses. Ensuring that these devices maintain their functionality over extended periods is crucial for their adoption in commercial and industrial settings.

### 2.3. Solvent-Assisted Molding

**Figure 5:**
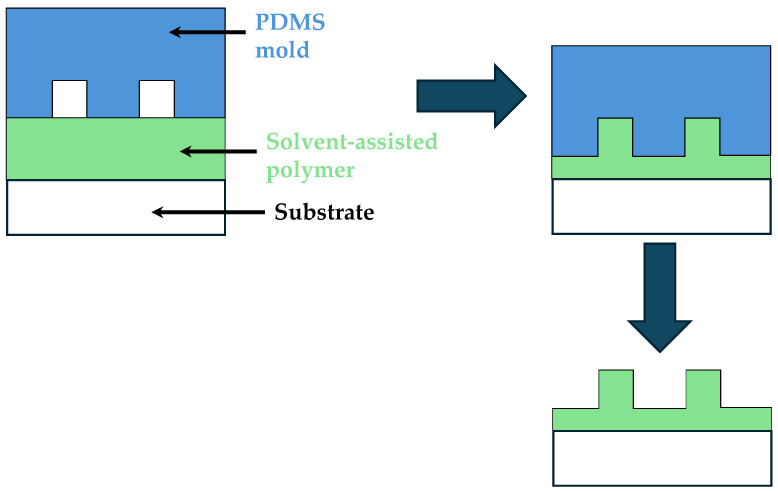
Schematic representation of the solvent assisted molding process.

Solvent-assisted molding enhances the fabrication process by using solvents to soften the polymer, making it more malleable for molding intricate designs. This technique is particularly beneficial for creating complex microfluidic structures that require high precision and detail [143,144,145]. The process, illustrated in Figure 5, involves applying a solvent to the polymer, which temporarily reduces its viscosity, allowing it to flow into the fine features of the mold. Once the solvent evaporates, the polymer solidifies, retaining the molded shape. This method is advantageous for materials that are otherwise difficult to mold, such as thermoplastics, and enables the fabrication of devices with intricate three-dimensional features [146,147,148].

Solvent-assisted molding offers several advantages, including the ability to mold high-aspect-ratio structures and complex geometries that are challenging for traditional molding techniques [149,150]. This method is particularly useful for fabricating microfluidic devices with integrated channels and reservoirs for chemical and biological assays. Applications include the development of lab-on-a-chip systems, microreactors, and diagnostic platforms. Solvent-assisted molding, specifically liquid composite molding (LCM) processes like resin transfer molding (RTM) and vacuum-assisted resin transfer molding (VARTM), offers the versatility to produce parts of complex shapes and features, providing a distinct difference from traditional molding techniques [151]. The use of a digitally reconfigurable surface in the precast and composite industry allows for a range of geometric outcomes from a single formwork, providing a novel approach that differs from traditional molding techniques [152].

Recent advances in solvent-assisted molding include the development of new solvent systems that enhance the molding process and improve the mechanical properties of the resulting devices. For instance, using a variety of green solvents, 2- and 3D designs containing nano- and micron-size structures on flat, vertical, and curved surfaces have been obtained [153], showcasing the potential for enhanced molding techniques. 

The use of solvent-assisted molding for fabricating multi-layered microfluidic devices with integrated optical and electronic components has also been explored. Solvent-assisted molding coupled with standard lithography enables the fabrication of intricate micro-flow networks and connection adapters, allowing for easy reconfiguration and the incorporation of components made from different materials [154]. Innovations such as the use of biodegradable polymers and bio-based solvents are expanding the environmental sustainability of this technique.

Nonetheless, while solvent bonding improves bonding strength, challenges related to surface roughness, microchannel morphology, and channel deformation need to be carefully addressed to ensure optimal device performance [145]. The use of solvent-assisted molding may require additional equipment, such as a 3D printer for mold construction, which should be considered in the fabrication process [155].

### 2.4. Injection Molding

**Figure 6:**
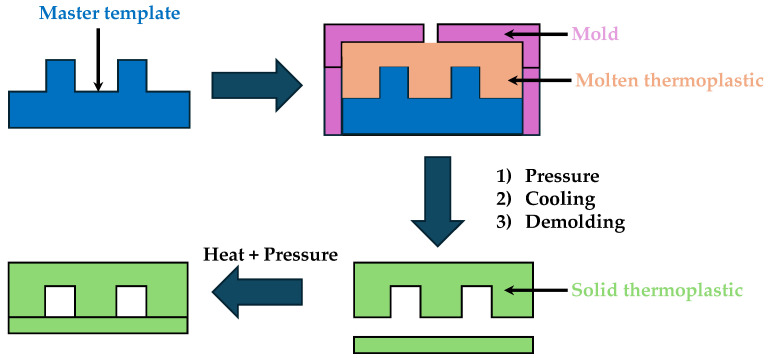
Schematic representation of the injection molding process.

Injection molding is a mass production technique where molten polymer is injected into a mold cavity under high pressure. This method is advantageous for producing large quantities of microfluidic devices with consistent quality and structural integrity. The process involves heating the polymer to a molten state then injecting it into a precision-engineered mold. Once the polymer cools and solidifies, the mold is opened, and the finished part is ejected. The process is schematically depicted in Figure 6.

Injection molding is particularly suitable for producing microfluidic devices with complex geometries and high aspect ratios, making it ideal for commercial applications where uniformity and scalability are critical [156]. 

Injection molding offers several advantages, including high production rates, excellent repeatability, and the ability to produce complex shapes with high precision. Injection molding allows for the production of high volumes of precise and complex components with high surface quality and dimensional accuracy [157]. The process offers high repeatability and process reliability, contributing to consistent part properties [158]. Injection molding is capable of mass-producing complicated plastic parts in a variety of complex shapes with high dimensional precision [159]. However, the initial cost of mold fabrication can be high, making it less suitable for small-scale production. Injection molding for low-volume production runs can be challenging due to long lead times and high costs associated with mold tooling [160]. Additionally, the choice of polymer is critical, as not all polymers are suitable for injection molding due to their thermal and rheological properties [161]. Injection molding process efficiency and part quality are influenced by part and mold design, machine and polymer selection, and process settings [162]. Mold temperature control impacts cooling time, surface quality, and inner structure of the molded parts. High-performance semi-crystalline polymers require high mold temperatures to achieve the desired structure, highlighting the importance of considering thermal properties in polymer selection [163]. In contrast with soft lithography, injection molding excels in scalability and is the preferred method for large-scale production of microfluidic devices. This technique allows for the rapid fabrication of thousands of identical devices with high precision. However, the initial setup costs for injection molding are significantly higher, and the process is less flexible when design modifications are needed. Thus, the choice between soft lithography and injection molding hinges on the specific needs of the project, whether it prioritizes prototyping efficiency or scalability.

Trends in injection molding include the development of micro-injection molding techniques that enable the production of microfluidic devices with sub-micron features. Advancements such as rapid injection molding and 3D-printed rapid tooling for injection molding are being explored to bridge the gap between prototyping and traditional injection molding, enabling faster turn-around time and cost reduction [164,165]. The use of advanced polymers, such as thermoplastic elastomers and high-performance engineering plastics, to improve the performance and durability of injection-molded microfluidic devices is also being explored. Thermoplastic microfluidic devices offer advantages over silicone elastomers and can be used to fabricate devices from a variety of plastics, ensuring high bond coverage, strength, optical clarity, durability, and low deformation or damage to microfeature geometry [166]. Thermoplastic elastomers can be processed with industrial polymer manufacturing technologies such as extrusion, injection molding, and hot embossing, allowing for scalable production and cheaper costs per part, impacting the fabrication process and design of microfluidic systems [167]. Thus, innovations such as the integration of micro-injection molding with other microfabrication techniques, such as hot embossing and laser micromachining, are expanding the capabilities of this method.

Challenges include difficulty in forming microstructures and residual stress, but optimization of process parameters can lead to high-quality microfluidic chips [168]. The replication of high-aspect-ratio micro features and uneven distribution of cavity pressure during mold filling remains as a partially unsolved demand [169,170].

### 2.5. 3D Printing

Three-dimensional printing has revolutionized the fabrication of microfluidic devices by enabling the direct creation of complex, multi-layered structures. Three-dimensional printing enables the rapid prototyping, device miniaturization, and customization of microfluidic devices with minimal fabrication steps [171,172]. It allows for the creation of complex 3D architectures with precise microstructures using functional materials and layer-by-layer assembly [172]. The technology offers cost-effective, time-efficient, and flexible fabrication methods for microfluidic systems [173,174].

Three-dimensionally-printed microfluidics have been applied in various fields, including point-of-care diagnostics, pharmacokinetic profiling, bacterial separation, and genotoxicity screening [175]. The technology has been used for the fabrication of microfluidic immunoarrays for ultrasensitive detection of multiple protein biomarkers, showcasing its potential in biomarker analyses [176]. Techniques such as stereolithography (SLA), fused deposition modeling (FDM), and digital light processing (DLP) allow for the customization of microfluidic devices with intricate geometries and integrated functionalities. SLA, for instance, uses a laser to cure photopolymer resin layer by layer, creating highly detailed structures. Two light-irradiation processes are commonly used: vector-by-vector laser-based irradiation and digital light projection [177,178]. FDM, on the other hand, extrudes a thermoplastic filament through a heated nozzle, building the device layer by layer. FDM typically uses thermoplastics like ABS, but there is a growing interest in high-performance materials such as PEEK. However, processing high-temperature materials presents new challenges, including the need to control outlet temperature for welding between layers and to ensure consistent mechanical properties [179,180,181]. DLP technology enables the fabrication of microchannels with cross-sectional dimensions as small as 20 μm × 20 μm [182]. The combined use of DLP and SLA 3D printing can create high-density microfluidic devices with active components such as valves, pumps, and multiplexers [183] and can enable extreme microfluidic component miniaturization and a high degree of component integration, including 3D-printed valves and integrated microfluidic elements for dose–response measurements [184]. Additionally, programmable pulsed aerodynamic printing (PPAP) is a versatile technique used in microfluidics for patterning soft materials with broad material compatibility and diverse properties. By using programmable pulsed airflow to shear droplets at the nozzle orifice, PPAP can precisely print droplets with various complex morphologies, including shell–core, Janus, and combined structures. Its co-flow configuration allows for accurate control across multiple scales and interfaces, making it a powerful tool for advanced material fabrication in microfluidic applications [185]. These methods offer unparalleled design flexibility, enabling the creation of microfluidic devices with complex internal channels and integrated components such as sensors and valves.

Three-dimensional printing offers several advantages, including rapid prototyping, design flexibility, and the ability to create complex structures that are difficult or impossible to fabricate with traditional methods. This technique is particularly useful for developing customized microfluidic devices for specific applications, such as organ-on-chip models, point-of-care diagnostics, and drug delivery systems. These devices are suitable for various biomedical applications, including cancer screening, drug testing, and point-of-care diagnostics [186,187,188]. They can be used for developing diagnostic microfluidic chips to detect analytes and biomarkers related to clinically relevant diseases [189]. The ability to integrate multiple functions into a single device, such as fluid handling, sensing, and actuation, enhances the versatility and functionality of 3D-printed microfluidic systems.

Recent developments in 3D printing include the use of multi-material printing techniques that enable the fabrication of devices with varying mechanical, chemical, and optical properties. Three-dimensional printing with multipurpose materials enables the production of advanced multifunctional polymer composites, offering mass customization, design freedom, and rapid prototyping capabilities [190,191]. The use of biocompatible and biodegradable materials for creating microfluidic devices for medical and environmental applications is also being explored [192,193,194]. Innovations such as the development of high-resolution 3D printers and the use of advanced post-processing techniques are enhancing the precision and performance of 3D-printed microfluidic devices [41,195].

The future of 3D-printed microfluidics may involve the evolution from 2D chips to 3D cubes, the integration of sensors and actuators during printing, and the rapid assembling of chips with printed microfluidic modules [196].

## 3. Materials Development

Polymers are integral to the development of microfluidic devices due to their versatility, ease of fabrication, and cost-effectiveness. The development of advanced polymer materials has played a crucial role in enhancing the functionality and expanding the capabilities of microfluidic devices. The integration of functional and smart polymer materials has been recognized as beneficial for the development of high-functioning microfluidic instruments, including stimuli-responsive hydrogels and conductive and magnetic composite polymers, which can realize electrodes, electronic routing, heaters, mixers, valves, pumps, sensors, and interconnect structures in polymer-based microfluidic systems [197]. Microfluidics offers a range of liquid templates for engineering materials with precise composition and morphology, establishing a basis for the meticulous control of wettability [198].

As indicated above, among the most commonly used polymers in microfluidics is polydimethylsiloxane (PDMS). Nonetheless, poly(methyl methacrylate) (PMMA) and cyclic olefin copolymers (COCs) have also been widely used as each of them offers distinct advantages and is suited to different applications. The main properties of PDMS have been introduced under previous sections. In contrast with PDMS, PMMA offers excellent optical properties, chemical resistance, and ease of fabrication through processes like injection molding [199,200]. These characteristics make PMMA a preferred choice for optical detection systems and applications where solvent resistance is critical [201,202]. However, PMMA’s brittleness and lower thermal resistance compared to other polymers can limit its use in more demanding environments [203].

Cyclic olefin copolymers (COCs) have emerged as a strong alternative to both PDMS and PMMA, particularly in applications requiring high chemical resistance and low water absorption. COCs provide excellent barrier properties, making them suitable for lab-on-a-chip devices and applications where moisture control is essential [204,205]. Additionally, COCs have superior dimensional stability and can withstand higher temperatures, which is advantageous in applications involving thermal cycling or where long-term durability is essential [206].

The choice of polymer for a microfluidic device should be guided by the specific requirements of the intended application. For example, in cell culture and biological assays, where optical clarity and biocompatibility are crucial, PDMS remains the material of choice despite its permeability issues. Conversely, for microfluidic devices used in chemical analysis or environmental monitoring, where exposure to harsh solvents or precise control over moisture is necessary, PMMA or COCs would be more appropriate due to their superior chemical resistance and lower permeability.

Furthermore, polycarbonate (PC) and polyethylene terephthalate (PET) are gaining attention for their use in microfluidic devices, particularly in applications requiring high mechanical strength and thermal resistance. Polycarbonate’s impact resistance and ability to withstand high temperatures make it suitable for devices exposed to mechanical stress or used in thermal cycling processes [207,208]. On the other hand, PET’s low cost and ease of processing make it an attractive option for disposable devices used in point-of-care diagnostics [209,210].

Ongoing research is focused on developing new polymer formulations and composites that combine the advantageous properties of existing materials while minimizing their limitations. For instance, fluorinated polymers are being explored for their exceptional chemical resistance and low surface energy, which can prevent nonspecific adsorption in analytical devices [211,212]. Additionally, the development of bio-based polymers is gaining traction as a sustainable alternative to traditional petrochemical-based materials, offering both environmental benefits and compatibility with biological systems [213,214].

Innovations in polymer science have led to the creation of materials with improved mechanical properties, chemical resistance, optical clarity, and biocompatibility. These advancements are critical for addressing the diverse and demanding requirements of modern microfluidic applications, ranging from flexible and wearable devices to robust and chemically resistant platforms. When selecting a fabrication method and material for a microfluidic device, it is crucial to consider the specific requirements of the intended application. For instance, while PDMS may be ideal for prototyping and applications involving cell cultures due to its biocompatibility, devices exposed to harsh chemicals or intended for mass production might benefit from alternative materials like COCs or the adoption of injection molding techniques. Researchers must weigh the advantages and disadvantages of each approach, considering factors such as chemical compatibility, scalability, cost, and the intended use case of the microfluidic device. This nuanced approach ensures that the chosen materials and fabrication methods align with the functional requirements and practical constraints of the project. 

This section explores the latest developments in polymer materials, highlighting their unique properties and contributions to the field of microfluidics. Although the manuscript primarily focuses on polymer-based devices, the unique advantages of glass capillary microfluidic chips in specific applications warrant attention. To provide a more comprehensive overview of the field, the following paragraphs include a brief discussion on these important devices. 

While polymer-based microfluidic devices dominate the field due to their versatility and ease of fabrication, glass capillary microfluidic chips represent a significant and valuable segment of microfluidic technology [215]. Glass offers several key advantages, including superior chemical resistance, excellent optical transparency, and high thermal stability. These properties make glass an ideal material for applications that involve aggressive solvents, high-temperature processes, or where precise optical measurements are critical, such as in fluorescence-based assays.

Glass microfluidic chips are particularly favored in situations where the inertness of the material is crucial to avoid any interaction between the chip and the reagents [216]. For instance, in analytical chemistry and certain types of biomedical diagnostics, the use of glass ensures that the microchannels do not react with the chemicals or biomolecules, thereby preserving the integrity of the results [217,218]. Additionally, glass chips can be easily cleaned and reused, which is advantageous in research settings where device longevity and reusability are important considerations.

Recent advances in microfabrication techniques, such as femtosecond laser machining and wet etching, have improved the precision and scalability of glass microfluidic chip production. These technologies have made it possible to create complex microchannel networks within glass substrates, enabling sophisticated fluidic control and analysis that were previously challenging to achieve with this material [219].

### 3.1. Flexible and Wearable Microfluidic Devices

The advent of flexible and wearable microfluidic devices represents a significant leap forward in personal health monitoring and diagnostics. The integration of microfluidics into wearable devices offers great potential for non-invasive monitoring and diagnosis of biofluids such as sweat, saliva, and tears [220,221,222,223]. Wearable microfluidic sensors have shown promise in assessing human health status, including monitoring sweat loss, metabolites, and electrolyte balance with high precision [222,224]. The development of miniature, flexible, transparent, and highly sensitive wearable sensors with microfluidic elements has enabled noninvasive and continuous monitoring of arterial blood pressure waveforms [224,225]. Materials such as silicone elastomers and fabrics are employed to create devices that conform to the human body, providing continuous monitoring of physiological parameters [226,227]. For example, PDMS is widely used due to its flexibility and biocompatibility, making it suitable for applications such as wearable sweat sensors and skin-interfaced health monitors [228]. These devices can track various biomarkers, including glucose, lactate, and electrolytes, offering real-time data that can be crucial for managing chronic diseases and optimizing athletic performance [229,230]. The integration of microfluidics with flexible substrates enables the development of unobtrusive and comfortable devices, paving the way for widespread adoption in personalized medicine. Implantable microfluidics and wearable microfluidic technologies have enormous potential in healthcare applications, including personalized diagnostics, targeted drug delivery, and biosensing for vital signs monitoring [224,231].

Flexible microfluidic devices are being developed for a range of applications, from healthcare to environmental monitoring [232]. For example, wearable sweat sensors can monitor hydration levels and detect metabolic disorders [233,234,235], while flexible patches can deliver drugs transdermally in a controlled manner [236,237,238]. Innovations such as stretchable electronics and flexible batteries are enhancing the functionality of these devices, enabling continuous monitoring and real-time data transmission [239,240]. The use of flexible microfluidic devices for environmental applications, such as monitoring air and water quality, is also being explored [33,34,232].

Challenges such as scalability and cost have been identified, leading to the exploration of alternatives such as thermoplastic elastomers (TPEs) for wireless, skin-interfaced devices [241]. Nonetheless, the field holds great promise for personalized medicine and predictive medical modeling [242,243].

### 3.2. Improved Mechanical Properties

The mechanical properties of polymers used in microfluidic devices are critical for their performance and durability. Recent advancements have focused on enhancing the tensile strength, elasticity, and toughness of these materials to withstand the rigorous demands of microfluidic applications. Polymers with high flexibility, good biocompatibility, and stiffness are widely used for microfluidic chip fabrication [22]. Photocured siloxanes have been successfully used to fabricate microfluidic devices for biomedical applications, demonstrating resistance to thermal cycles and limited water swelling [19]. The stiffness of microcapsules, for instance, can be tuned by adjusting the thickness and cross-link ratio of the polymer shell, enabling the design of elastic microcapsules tailored for specific flow behavior in various applications [244]. Thermoplastic polymers, such as poly(methyl methacrylate) (PMMA) and polycarbonates (PC), offer durability and resistance to high shear stress conditions, making them suitable for microfluidic applications [21]. Thermoplastic elastomers such as polyurethane (PU) and polycarbonate (PC) have been developed with improved mechanical properties, ensuring the durability and reliability of microfluidic devices in dynamic and high-stress environments [245,246]. These materials can endure repeated mechanical deformation, making them ideal for applications such as portable diagnostic devices and lab-on-a-chip systems that require robust and long-lasting components.

Research in this area has led to the development of new polymer blends and composites that offer enhanced mechanical properties. For example, incorporating nanofillers such as carbon nanotubes and graphene into polymer matrices can significantly improve their strength and toughness [247,248]. The use of bio-based and biodegradable polymers, which offer environmental benefits while maintaining excellent mechanical performance, is also being explored [22,249,250]. The development of polymers with self-healing properties is another exciting area of research [251,252]. These materials have the ability to restore their original strength and recover their inherent properties when mechanically damaged, without the need for human intervention [253]. Self-healing polymers can be classified into extrinsic and intrinsic materials, with extrinsic materials having the repairing agent pre-embedded in the resin matrix, while intrinsic materials do not have an embedded healing agent and require an external stimulus to initiate the healing process [254]. Thus, these materials have the potential to create microfluidic devices that can repair themselves after damage, extending their lifespan and reliability [255].

Regarding the challenges that remain in connection with the development of materials with enhanced mechanical properties, it is worth noting the hydrophobic nature and low surface energy of some polymers, and particularly PDMS, which handicaps the bonding with other polymers, affecting mechanical properties [49]. Also, specific surface modification and functionalization steps are required to tailor the surface chemistry of polymer-made channels, impacting mechanical properties [18]. For instance, to address the limitations of PDMS, various surface modification techniques have been explored. For example, coating PDMS with materials such as parylene or employing plasma treatments can significantly reduce its permeability to gases and solvents, thereby enhancing its performance in microfluidic applications where these properties are critical. Additionally, alternative materials like cyclic olefin copolymers (COCs) and thermoplastics are gaining traction due to their superior chemical resistance and lower gas permeability. These materials, while less flexible and more challenging to work with than PDMS, offer a viable solution for applications requiring greater chemical stability and durability. The selection of polymer microfabrication is crucial for successful microfluidic applications, with PDMS and thermoplastic materials offering unique advantages [17].

### 3.3. Enhanced Optical Properties

Microfluidic devices often rely on optical detection and imaging techniques, necessitating materials with high transparency and low autofluorescence. Advanced polymers with these properties have been developed to facilitate accurate and efficient optical analysis in microfluidic systems. For example, cyclic olefin copolymer (COC) and cyclic olefin polymer (COP) are highly transparent and exhibit excellent optical clarity, making them suitable for applications involving fluorescence microscopy and spectrophotometry [205,256,257]. These materials allow for precise observation and measurement of biological and chemical processes within microfluidic channels, enhancing the sensitivity and specificity of analytical assays. To confer enhanced optical properties to polymers, different strategies have been developed. Recent innovations include the development of polymers with tunable optical properties, such as adjustable refractive indices. Fluorination of polymers can reduce the refractive index, making it similar to that of water, which is beneficial for optical measurements in microfluidic devices [258]. The addition of titanium dioxide to poly(dimethyl)siloxane (PDMS) can enhance light reflection properties, increasing the collected light from fluorescent and luminescent moieties inside microfluidic channels by up to 11-fold, thus improving sensitivity and lowering detection limits [259]. Thiol–ene polymers offer rapid UV curing, low volume shrinkage, and optical transparency, making them suitable for use in microfluidic devices [260]. These materials enable the creation of microfluidic devices with integrated optical components, such as lenses and waveguides, for advanced analytical applications. Microscale diffractive lenses composed of aperiodically spaced concentric rings milled into a thin metal film have been presented as a method by which to position optical tweezers within microfluidic channels precisely [261]. They are used as photoluminescent, photochromic, photocleavable, and photocross-linkable polymers. These polymers are designed with photosensitive moieties to enable reversible, irreversible, and dynamic responses to light irradiation [262]. Photoresponsive polymers have potential applications in microfluidic devices for controlled drug delivery and optical signal modulation [263,264]. They enable dynamic regulation of biological interactions and cellular behaviors in response to light, offering spatiotemporal control of biological processes [263].

Challenges are often related to fabrication techniques. Techniques such as micro-mixing and flow-focusing in microchannels can be utilized for the rapid and stable fabrication of microfluidic devices for ferroelectric polymers’ synthesis, offering a flexible platform for various applications [265]. The fabrication of microfluidic optical cells using a micropatterned polymer mold and imprinting on thermoplastic substrates has also been introduced, resulting in improved sensitivity and reduced noise for optical measurements [266]. Other challenges in the field include moving from harmful UV light to visible/near IR light and enabling biomedical applications. Future opportunities include developing light-controlled supramolecular actuators and multi-stimuli-responsive supramolecular systems [267].

### 3.4. Superior Chemical Resistance

Chemical resistance is a critical factor for microfluidic devices used in harsh chemical environments. Recent advancements have led to the development of polymers with enhanced resistance to solvents, acids, and bases, extending the range of applications for polymer-based microfluidic devices. For instance, perfluoroalkoxy alkane (PFA) and fluorinated ethylene propylene (FEP) exhibit excellent chemical resistance, making them suitable for applications involving aggressive reagents and solvents [268]. These materials ensure the integrity and longevity of microfluidic devices, enabling their use in chemical synthesis, environmental monitoring, and pharmaceutical applications [269,270]. 

The development of polymers with improved chemical resistance involves the synthesis of new monomers and the modification of polymer structures to enhance their stability. For example, incorporating fluorine atoms into polymer backbones can significantly increase their resistance to chemical attack. Thermoplastic polymers such as high-density polyethylene (HDPE), polyvinylchloride (PVC), and poly(vinylmethylsiloxane) (PVMS) networks have demonstrated excellent chemical resistance for use in microfluidics [21,50,271]. HDPE microfluidic devices have shown excellent compatibility with a range of organic solvents, making them suitable for chemical reactions in aromatic and hydrocarbon solvents [50]. PVC microfluidic devices exhibit drastically reduced gas permeability compared to polydimethylsiloxane (PDMS), expanding their range of applications [50]. Surface treatments and coatings that provide additional protection against harsh chemicals is also an option that further extends the lifespan and reliability of microfluidic devices. For instance, surface modification of poly(dimethylsiloxane) (PDMS) using perfluoroalkane–triethoxysilanes has been shown to enhance its resistance to organic solvents, making it suitable for specific microfluidic applications [272]. Thiol–ene materials have been shown to be more solvent resistant than most other commonly used polymers, resulting in exceptional solvent compatibility, even in challenging chemical environments [273].

Some challenges and limitations associated with the search for polymers with superior chemical resistance are yet related with specific surface modification and functionalization steps to tailor the surface chemistry of microfluidic devices with respect to the desired application, posing a challenge in the fabrication process [18]. Also, the integration of polycarbonate track-etched (PCTE) membranes with polydimethylsiloxane (PDMS) microfluidic devices presents challenges due to the hydrophobic nature and low surface energy of PDMS, impacting the reproducibility of the integration process [274]. While fluorinated materials are chemically inert and resistant to organic solvents, only a few resin formulations have been demonstrated suitable for 3D printing chemically resistant polymer objects [275]. A simple yet effective treatment of thiol–ene materials through a temperature treatment results in exceptional solvent compatibility, even for very challenging chemical environments, such as chlorinated solvents [273,275]. Finally, homemade resin formulations based on perfluoro-1,6-hexyl diacrylate (PFHDA) for high-resolution 3D printing utilizing micro-stereolithography have been introduced as a potential solution for fabricating chemically resistant polymer objects for microfluidics applications [275].

### 3.5. Electrical Conductivity

Incorporating electrically conductive materials into microfluidic devices has opened new avenues for integrated electronics and sensors. Conductive polymers and composites are being used to create microfluidic devices with embedded electrodes and circuits, enabling sophisticated functionalities such as electrochemical sensing and actuation. For example, polyaniline (PANI) and poly(3,4-ethylenedioxythiophene) (PEDOT) are conductive polymers that can be patterned within microfluidic channels to create electrochemical sensors for detecting biomolecules, ions, and other analytes. These polymers are promising for interfacing with biological organisms [276]. PEDOT/PANI-based copolymer using electrochemical oxidative polymerization has proven to be successful in the formation of conductive polymers onto a glass substrate [277]. PANI alone has been used as a sensor for detecting dissolved ammonia, showing high sensitivity and repeatability [278], whereas PEDOT has been enhanced through covalent modification, resulting in improved adhesion to substrates and better electrochemical properties, making it suitable for biological sensing applications [279]. Various thin-film deposition techniques, such as roll-to-roll printing and spin coating, have been employed to fabricate PANI and PEDOT-based sensors [280]. PEDOT-based materials have been fabricated with large surface area, high conductivity, and good biocompatibility, making them suitable for electrochemical sensing applications in environmental monitoring, food and drug analysis, and health care [281]. The integration of conductive materials with microfluidics enhances the versatility and functionality of these devices, allowing for the development of advanced diagnostic and analytical tools [282]. Fast, simple, and cost-effective techniques for integrating electrodes into thermoplastic microfluidic chips using an off-the-shelf conductive ink have been developed, enabling rapid prototyping of microfluidic devices for capacitance sensing, droplet merging, and sorting [283]. Some designs integrate smart electronics and microfluidics in an elastomer package, allowing for the precise delivery of liquid samples to the integrated circuits and enabling compact flexible electronic and lab-on-a-chip systems for applications such as wearable health monitoring and point-of-care diagnostics [284].

Recent innovations include the development of stretchable and flexible conductive polymers, which can be integrated into wearable and flexible microfluidic devices. The fabrication of stretchable devices is typically achieved through the use of stretchable polymer-based conductors or more rigid conductors with patterned geometries that can accommodate stretching. Other approaches, such as metallization pattern stamping, have been developed to enable the integration of stretchable interconnects with wearable fabrics and the creation of fully-flexible electromagnetic microactuators [285]. These materials enable the creation of devices that can conform to the body and provide continuous monitoring of physiological parameters. The use of nanocomposites, such as graphene and carbon nanotube composites, to enhance the electrical conductivity and mechanical properties of microfluidic devices is also being investigated. Carbon-based nanomaterials like graphene and carbon nanotubes, when added to a polymer material, can form conductive composites, allowing free electrons to travel easily and conduct electricity [286]. These conductive composites are lightweight, corrosion-resistant, and adaptable to specific applications, making them suitable for replacing metals in certain applications [286]. The electrical conductivity of nanocomposites is influenced by factors such as filler loading, agglomeration, and the aspect ratio of the fillers [287,288,289]. Nanocomposites containing graphene and carbon nanotubes also exhibit enhanced mechanical properties, including improved tensile strain, piezoresistive, and thermoresistive sensitivities [290]. The addition of graphene and carbon nanotubes to polymers can enhance their mechanical properties, allowing for the creation of high-strength, low-density materials with improved conductivity [291,292]. Challenges in the use of nanocomposites, such as graphene and carbon nanotubes, include issues related to nanoparticle agglomeration, dispersion, and viscosity, which can affect processing and load transfer in the composites [292,293]. Additionally, the functionality of nanocomposites depends on overcoming challenges such as the breakdown of nanoparticle agglomerates, attachment of functional materials to nanoparticle surfaces, and fine dispersion within polymeric matrices [293]. The incorporation of carbon nanomaterials in microfluidic devices has opened up new opportunities for improving the figures of merit in the final analysis, leading to low-volume, rapid, and simple analysis [294].

The development of bio-compatible conductive materials is another exciting area of research, with the potential to create microfluidic devices for in vivo applications, such as implantable sensors and drug delivery systems. Microfluidic devices with integrated electrical sensors have been widely employed in the detection and characterization of particles suspended in liquids, showcasing potential applications in biomedical sectors such as drug delivery, diagnosis devices, cell culture, and scaffold fabrication [295,296]. Integration of microfluidics and electrochemical (bio)sensors is envisioned as a powerful tandem for boosting the next generation of lab-on-a-chip platforms, including point-of-care and point-of-need systems, indicating potential applications in industrial domains and commercialization [297].

## 4. Advanced Fabrication Techniques

Advances in fabrication techniques have been instrumental in pushing the boundaries of what is possible with microfluidic devices. The ability to create intricate and precise micro-scale features is essential for the development of sophisticated microfluidic systems. Advanced fabrication methods such as nanoimprint lithography, laser micromachining, and hybrid fabrication techniques have enabled the production of complex, high-resolution structures with integrated functionalities [298,299,300]. These techniques not only enhance the performance and reliability of microfluidic devices but also expand their potential applications. This section delves into the latest advancements in fabrication technologies, examining their principles, advantages, and the novel capabilities they bring to the field of microfluidics.

### 4.1. Nanoimprint Lithography

**Figure 7:**
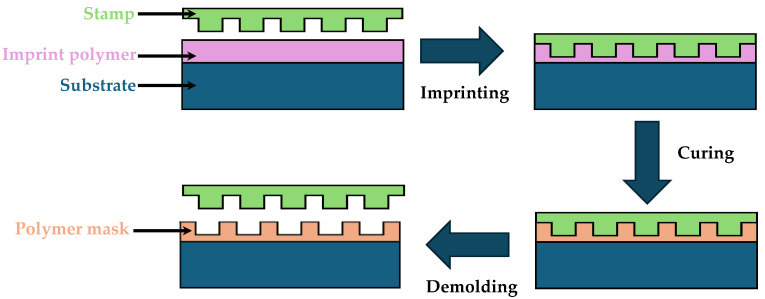
Schematic representation of the nanoimprinting lithography process.

Nanoimprint lithography (NIL) is an advanced technique for fabricating nanoscale patterns on polymer surfaces. NIL involves pressing a hard mold with nanoscale features into a polymer film, which is then cured to transfer the pattern, as schematically illustrated in Figure 7. This method is highly effective for creating high-resolution features with excellent uniformity.

Nanoimprint lithography (NIL) has various applications in nanofluidics, including the fabrication of micro/nano-fluidic devices for biomedical applications [301,302,303]. It is used in the fabrication of microchannel molds with nanopatterns to control microfluidic behavior, which is crucial for applications in nanofluidics [304]. NIL contributes to the development of biosensors, such as nanostructured plasmonic biosensors and biosensors based on nanoelectromechanical systems, which are essential for nanofluidic applications [302]. The technique is also employed in tissue engineering, particularly in guiding cells cultured on micro- or nanostructured substrates [302].

NIL contributes to the development of nanofluidic devices by enabling the fabrication of unique nanoscale devices for various applications, including optics, plasmonics, and biotechnology. Recent advances in throughput and yield in NIL processes demonstrate the potential of adopting NIL for mainstream semiconductor device fabrication, including nanofluidic devices [305]. Recent developments in large-area nanoimprint lithography have made it a unique technology for fabricating micro/nano optical and optoelectronic devices, including microfluidic devices, which are crucial for nanofluidic applications [301]. High-volume nanoimprint lithography has been introduced, requiring well-tuned processes and materials, and has been tested in industrial environments for applications such as high-brightness light-emitting diodes (HBLEDs) [306]. Improvements in nanoimprint lithography alignment systems and high-order distortion correction (HODC) systems have enabled better distortion and overlay results, making it suitable for advanced memory applications with tight overlay budgets [307,308].

One of the limitations of current NIL techniques is the lack of flexibility in patterning, which can pose challenges in certain nanofluidic applications [309]. While NIL offers high throughput and cost-effectiveness, it faces challenges in achieving large-scale 3D fabrication capability with resolutions towards 10nm or less, which is essential for nanofluidic devices [309].

### 4.2. Laser Micromachining

Laser micromachining uses focused laser beams to ablate material and create micro-scale features on polymer surfaces. This technique is highly precise and can create complex geometries with high aspect ratios. Laser micromachining is particularly useful for fabricating microfluidic devices with intricate channel networks and integrated optical components.

Laser micromachining offers several advantages, including high precision, flexibility in design, and the ability to process a wide range of materials. This technique is used for creating microfluidic devices for applications such as cell sorting, chemical analysis, environmental monitoring, and microfluidic devices [310,311]. Laser micro/nanomachining technology has been applied to fabricate spherical structures of soft matter, showcasing its potential for nanofluidics and biomedicine [312]. The process parameters, such as power, machining speed, number of passes, and laser focus distance, are varied to create microchannels with specific surface qualities and dimensions [310,311]. The laser energy and pulse rate affect the depth of micromachining channels, while rectangular variable aperture (RVA) in both x- and y-directions affects the width of the channels [313]. Proper adjustment of laser energy and pulse rate is required to fabricate desired channel depths [313]. 

Some types of laser micromachining include the following:

- Femtosecond laser micromachining: Utilizes ultrashort laser pulses to ablate material with minimal heat-affected zones, ideal for creating precise and intricate features in microfluidic devices. Femtosecond laser micromachining has been used for manufacturing micro- and nanofluidic devices, indicating its relevance in nanofluidics applications [314,315,316].
- Excimer laser micromachining: Uses ultraviolet lasers to achieve high-resolution patterning on polymers, suitable for microfluidic device fabrication with complex geometries [313].
- CO_2_ laser micromachining: Effective for cutting and engraving polymer substrates, often used in the initial stages of microfluidic device fabrication for rapid prototyping [317].

Recent advancements include the use of femtosecond lasers, which offer even higher precision and minimal thermal damage to the material, enabling the fabrication of ultra-fine features. Laser micromachining offers a promising alternative method for rapid production of microfluidic devices, but the effect of process parameters on the channel geometry and quality of channels on common microfluidic substrates has not yet been fully understood [311]. This technique has been used as an alternative to producing microfluidics structures and simplifying the conventional soft lithography process [313]. Hybrid laser electrochemical micromachining is a type of micromachining that combines the advantages of laser and electrochemical machining techniques, offering high precision, quick machining speed, low thermal stress, and high material removal rate [318]. It has also emerged as a promising technique for mass production of microfluidic devices, and it offers high flexibility in channel dimensions and morphology by controlling the laser properties [319]. 

Other innovations in laser micromachining include the development of hybrid techniques that combine laser ablation with other micromachining methods, such as chemical etching and mechanical milling [320,321,322]. These hybrid approaches enhance the precision and versatility of the fabrication process, enabling the creation of more complex microfluidic devices. Systematic studies have been conducted to understand the effect of laser system parameters and thermophysical properties of substrate materials on laser micromachining, providing insights into the optimization of process parameters for microfluidic devices [323]. Studies on the role of a focused laser in micro- and nanofluidic systems are being widely introduced with special interest in thermofluid dynamical aspects and their importance in optical manipulation [324].

Current challenges in the application of laser micromachining in nanofluidics include controlling and optimizing process parameters, designing substrate materials, and achieving high surface quality [323]. The effect of process parameters on the channel geometry and quality of channels on glass substrates has not yet been fully understood [325]. Also, the difficulty in translating conventional microfluidics from laboratory prototypes to commercial products has shifted research efforts towards thermoplastic materials for their higher translational potential and amenability to industrial manufacturing [319]. Future directions involve the integration of real-time monitoring and feedback systems to optimize the micromachining process, ensuring high-quality and consistent results.

### 4.3. Hybrid Fabrication Techniques

Hybrid fabrication techniques combine multiple fabrication methods to create complex and multifunctional microfluidic devices. These techniques leverage the strengths of different methods to achieve desired features and functionalities that are challenging to obtain with a single technique. Hybrid fabrication techniques in microfluidic device manufacturing involve combining multiple methods such as 3D printing, photolithography, and xurographic technique [326,327,328,329]. These techniques enable the creation of complex and multifunctional microfluidic devices by seamlessly integrating a broad range of structural and functional materials into the devices [326,327,328,330].

The use of multi-materials multi-scale hybrid printing allows for the fabrication of microfluidic biosensors with embedded fluidic channels and functionalized electrodes at sub-100 µm spatial resolution, demonstrating sensitive response and a linear dynamic range relevant to physiological levels of analytes in sweat [326]. Additionally, the combination of conventional photolithography and two-photon polymerization has been shown to form a simple hybrid approach in fabricating master molds for soft lithography, benefiting from the convenience of photolithography and complex 3D structures with high resolution based on two-photon polymerization [80]. Furthermore, a novel, cost-effective, hybrid microfluidic chip manufacturing technology has been proposed, combining the 3D printing process and the xurographic technique, demonstrating potential applications in biomedicine and material science [327]. The integration of functional and/or sensing materials in microfluidic devices has been achieved through the combination of the conventional 3D printing fabrication process with the stable and precise integration of polymeric functional materials in small footprints within the microchannels, enhancing the adhesion force between the microstructures and the 3D-printed microfluidic device [330].

Examples of hybrid fabrication techniques include the integration of injection molding with laser micromachining to fabricate high-throughput devices with precise features. These hybrid techniques are used for developing advanced lab-on-a-chip systems, wearable sensors, and implantable devices. Some of the most widely used hybrid fabrication techniques are briefly discussed in the following paragraphs. The combination of 3D printing and soft lithography has been explored in various studies [155,331,332,333]. The use of 3D-printed molds for fabricating multi-layer PDMS-based microfluidic devices has been demonstrated, showcasing the potential for integrating 3D printing with soft lithography [155].

Focused ion beam (FIB) micromachining is a high-precision technique that uses a focused beam of ions to remove material at the micro- and nanoscale. This method is particularly useful for creating detailed microstructures and modifying existing microfluidic devices. FIB micromachining is used for applications such as creating microchannels, vias, and reservoirs in microfluidic devices. It is also employed for modifying and repairing microfluidic structures with high precision. FIB is a powerful tool for maskless lithography and strain engineering, enabling the fabrication of freestanding thin film structures with large lateral dimensions [334]. It can be used for direct writing/patterning of various materials, creating a variety of geometric features without the need for masks [335]. FIB can be utilized for micro/nanofabrication, including milling, deposition, and surface self-organization processes, making it a multipurpose tool [336,337,338].

FIB has applications in failure analysis, circuit modification, and semiconductor device fabrication, making it a valuable tool for industrial purposes [336,339]. Innovations in FIB technology include the development of dual-beam systems that combine FIB with scanning electron microscopy (SEM) for real-time imaging and precise control of the micromachining process. Advancements in FIB processing systems, such as the development of automatic processing and recognition, pave the way for mass production of nanoholes with high precision and speed [340]. One limitation of FIB micromachining is the residual stresses formed during processing, affecting the fabrication of flat freestanding thin film structures [334]. Research has been conducted to investigate the optimum parameters for milling microchannels, addressing issues such as channel width, gap, and depth [335].

Electrochemical micromachining (ECMM) uses electrochemical reactions to remove material and create micro-scale features on metallic and polymer substrates. ECMM is based on the principle of electrolysis, where the workpiece acts as an anode and the tool as a cathode, and material removal occurs via anodic dissolution at the atomic level [341]. The process operates at the smallest inter-electrode gap (IEG) with pulsed voltage as input for localized material dissolution to fabricate microfeatures. This technique is highly selective and can achieve high precision without inducing thermal or mechanical stress. ECMM is used for fabricating microfluidic devices with features such as microchannels, nozzles, and electrodes. The process involves the use of conductive materials to create complex features on the workpiece [342]. The process parameters, such as voltage, interelectrode gap, machining time, duty cycle, and electrolyte concentration, have been studied for fabricating micro tools and patterns, providing insights into the process optimization [343,344]. Passivation layer formation is a challenge in ECMM, affecting the accuracy of machined holes, but an electrolyte flushing technique has been shown to improve circularity by effectively removing the passivation layer [342]. It is particularly advantageous for machining hard-to-machine materials and for applications that require high aspect ratio features. ECMM is also used for surface modification and functionalization of microfluidic devices. 

Some challenges of hybrid fabrication techniques include limitations in achievable design complexity, the need for a wider variety of transparent materials, limited z-resolution, and absence of extremely smooth surface finish [328]. The influence of fabrication parameters, materials, and bonding layers on the channel dimensions, performances, and durability in the process of chip realization also constitute potential challenges in optimizing these parameters [327]. Limitations in precision fabrication of hollow and void sections with an extremely high surface-area-to-volume ratio, limitations in achievable z-resolution, and extremely smooth surface finish must also be taken into consideration [328]. 

### 4.4. Emerging Materials

Due to the importance of this research filed, the development of new materials aimed at microfluidics is drawing the attention of the scientific community; thus, a wide variety of emerging materials are being investigated nowadays.

Biodegradable polymers are gaining attention for their potential to create environmentally sustainable microfluidic devices. These materials can degrade into non-toxic byproducts after their intended use, reducing environmental impact and enabling applications in transient electronics and medical implants. Biodegradable polymers, both natural and synthetic, are being widely used for various applications, including microfluidic chip fabrication and tissue engineering [22,345]. Recent advances in microfluidic technology have highlighted the use of polymers to construct microfluidic scaffolds and generate uniform particles for drug delivery and artificial cells. The combination of biodegradable polymers and microfluidics presents a low-cost, reproducible, and scalable approach for manufacturing biomimetic scaffolds for tissue engineering [22]. Biodegradable polymers are essential for reducing pollution and the impact on human health, making them a potential solution to environmental issues related to polymers [346,347,348]. These materials have applications in drug delivery, tissue engineering, wound healing, and biosensors, showcasing their versatility and potential in the biomedical field [348,349]. Biodegradable microfluidic devices are being developed for applications such as drug delivery, tissue engineering, and environmental monitoring. Innovations in this field include the development of biodegradable elastomers with improved mechanical properties and the use of natural polymers, such as chitosan and alginate, for biocompatible and degradable microfluidic systems [350].

Stimuli-responsive polymers can change their properties in response to external stimuli, such as temperature, pH, or light. These materials offer dynamic control over microfluidic processes, enabling the development of smart devices that can adapt to changing conditions. Stimuli-responsive polymers are integrated into microfluidic devices to achieve controlled fluid delivery, actuation, and manipulation at the microscale [351]. They are utilized as passive and active fluid control elements, enabling functions such as gate and valve operations, pumping, and flow manipulation [352]. Stimuli-responsive microfluidic devices are being developed for applications such as controlled drug delivery, chemical sensing, and adaptive diagnostics. Current challenges include improving switching times and achieving local addressability of the responsive constituent, whereas future directions involve utilizing redox-responsive polymers for fast and reversible switching and local addressability in combination with nanofabricated electrodes. Innovations include the development of polymers that can change their wettability or permeability in response to stimuli, enabling dynamic control of fluid flow and reagent mixing within microfluidic channels.

Conductive hydrogels combine the flexibility and biocompatibility of hydrogels with electrical conductivity. These materials are used to create bio-interfaced microfluidic devices that can interface with biological tissues and provide electrical stimulation or sensing capabilities. Conductive hydrogel-based microfluidic devices are being developed for applications such as neural interfaces, biosensors, and tissue engineering. Innovations include the development of hydrogels with tunable conductivity and mechanical properties, enabling the creation of devices that can conform to complex biological structures and provide precise electrical control.

## 5. Applications of Polymer-Based Microfluidic Devices

Polymer-based microfluidic devices have found a wide range of applications across various fields due to their versatility, cost-effectiveness, and ease of fabrication. These devices have revolutionized numerous domains by enabling precise fluid manipulation, high-throughput analysis, and integration with other technologies. From biomedical applications such as diagnostics [353,354], drug delivery [355,356,357], and tissue engineering [358,359,360] to environmental monitoring [361] and industrial processes [362,363], polymer-based microfluidics offer innovative solutions to complex challenges. This section explores the diverse applications of these devices, highlighting their impact and potential in transforming research and industry practices.

### 5.1. Biomedical Applications

As stated above, polymer-based microfluidic devices have numerous applications in the biomedical field, including diagnostics, drug delivery, and tissue engineering. Microfluidic devices are being used to develop point-of-care diagnostic tools that can rapidly detect diseases and monitor health conditions [364,365,366]. These devices can perform complex assays with small sample volumes, providing quick and accurate results. Microfluidic devices are used to create controlled drug delivery systems that can release drugs at specific rates and locations. These systems are being developed for applications such as targeted cancer therapy [367,368] and chronic disease management [369]. Microfluidic devices are used to create three-dimensional tissue models that mimic the structure and function of human tissues. These models are used for studying disease mechanisms, testing drugs, and developing regenerative medicine therapies.

#### 5.1.1. Organ-on-a-Chip (OoC) Technology

OoC is a microfluidic device that models organs and organ systems, offering high efficiency and accuracy for disease or drug testing models. OoC devices rely on microfluidic principles to model cell and tissue environments in minuscule volumes, often using 3D printing for increased complexity and faster prototyping [370]. These devices simulate tissue and organ-level physiology, enabling high-resolution, real-time imaging and in vitro analysis of biochemical, genetic, and metabolic activities [371]. OoC has potential applications in drug target optimization, drug screening, toxicity testing, and biomarker identification [372,373]. Microfluidic technology allows for precise fluid control and has been widely applied in OoC systems to mimic specific organs or multiple organs in vivo [374]. OoC platforms have been developed for various organs such as blood vessels, lung, liver, and skin, offering benefits in toxicity screening, target discovery, and potentially replacing animal testing [375,376]. The integration of artificial intelligence has revolutionized organoid imaging, enhancing high-throughput drug screening in OoC systems [377]. Polymers such as PDMS are commonly used due to their flexibility and biocompatibility.

Liver-on-a-chip devices are used to study liver function, drug metabolism, and toxicity [378]. They provide a more accurate representation of human liver physiology compared to traditional cell culture models [379,380]. Heart-on-a-chip models simulate cardiac tissue, enabling the study of heart function, disease mechanisms, and drug effects [381,382]. They are particularly useful for testing the cardiotoxicity of new drugs [383]. The integration of multiple organ models into a single device, known as a “body-on-a-chip”, represents the next frontier in OoC technology [384]. This approach aims to recreate the interactions between different organs, providing a holistic view of human physiology and disease [385,386].

#### 5.1.2. Microfluidics in Personalized Medicine

Personalized medicine aims to tailor medical treatment to the individual characteristics of each patient. These devices can process small samples with high accuracy and speed, making them ideal for personalized medicine applications, enabling medical decisions and interventions based on individual patients’ predicted response or disease risk [27]. Microfluidics is involved in the diagnosis and therapy of various diseases, including cancer, cardiovascular disease, diabetes, and pulmonary disease, using biomarkers and assay-based methods [387]. Microfluidic biochips are promising for generating different concentrations of personalized medicine, addressing the need for specific drug concentrations tailored to individual patients [388]. Microfluidic droplet technology offers new ways to measure and detect biomolecules, facilitating high-throughput screening of biomarkers and providing miniaturized diagnostic systems for personalized medicine [389,390]. Accurate and efficient control of droplet generation is essential for the successful application of droplet microfluidics in areas such as material synthesis and lab-on-a-chip systems [391].

Microfluidic devices are used for genetic analysis, including DNA sequencing, gene expression profiling, and genotyping [392]. For instance, microfluidic devices are used to analyze tumor samples, identifying genetic mutations and guiding personalized cancer treatment [393,394]. Also, these devices can determine how a patient’s genetic makeup affects their response to drugs, enabling the selection of the most effective and least toxic treatments [395].

Despite challenges and limitations, microfluidics has the potential to greatly impact the pharmaceutical industry, improving drug discovery, development, manufacturing, and point-of-care testing for personalized medicine [396,397].

#### 5.1.3. Drug Screening

Microfluidic devices are used to screen drugs on patient-derived cells and tissues, allowing for the identification of the most effective treatments for individual patients. This approach reduces the risk of adverse effects and improves treatment outcomes [398]. Three-dimensional microchips can recapitulate various biochemical and biophysical conditions in vivo, providing versatile in vitro models for drug screening. Furthermore, bioassays on microfluidics can avoid ethical issues related to experiments on animals or humans, which can be a significant obstacle in conventional drug screening methods [399]. Microfluidic platforms also offer a faster and less-expensive alternative for drug screening, with the ability to mimic in vivo cell systems in a simple and inexpensive manner [27]. Microfluidic-based high content screening (HCS) reduces experimental costs, increases assay throughput, and improves the accuracy of drug screening, making it a promising technology for drug discovery and screening [400]. Microfluidic devices enable the recreation of physiologically relevant cell culture conditions, improving the screening of drug candidates prior to in vivo testing [401]. Finally, microfluidic cell chips integrate 3D cell culture, providing benefits such as reduced sample quantity and more representative physiological/pathological microenvironments for drug screening [402]. The integration of microfluidic devices with other technologies, such as artificial intelligence and big data analytics, is expected to further advance personalized medicine. These innovations will enable the development of more precise and effective therapies tailored to the unique characteristics of each patient. However, it is important to note that while microfluidic devices offer significant potential for drug screening, routine use in ordinary labs may be limited due to the complexity involved in device fabrication and system setup [403].

### 5.2. Environmental Monitoring

Polymer microfluidic devices offer advantages over traditional laboratory-based techniques such as miniaturization, portability, cost-effectiveness, and ease of use, making them suitable for environmental monitoring [404,405]. These devices offer rapid response capabilities, making them suitable for continuous environmental monitoring, including air, water, soil, and agricultural matrices [406]. Also, they provide a more robust and durable platform compared to paper-based microfluidic devices, making them suitable for long-term environmental monitoring [407]. Various detection methods, including colorimetric, fluorescent, and electrochemical methods, have been studied for environmental monitoring using microfluidic devices. Also, the use of threads for solid state reagent storage in polymer-based microfluidic devices enables autonomous measurement with minimized energy cost [408]. Advancements in micromilling parameters have led to improved surface quality of polymer microfluidic devices, making them suitable for rapid prototyping and testing of environmental monitoring devices [404].

Polymer-based microfluidic devices are used for monitoring environmental conditions, such as air and water quality [409]. These devices can detect pollutants, pathogens, and other contaminants with high sensitivity and specificity. Devices are being developed to detect contaminants in water, such as heavy metals, bacteria, and organic pollutants, enabling the provision of real-time monitoring and rapid response to environmental hazards [410,411]. Microfluidic devices are also used to monitor air quality by detecting pollutants, such as particulate matter, volatile organic compounds, and gases [412,413]. These devices can be integrated into portable sensors and wearable devices for continuous monitoring of air quality.

Recent advancements in the field of microfluidic devices for environmental monitoring highlight the potential for continuous and autonomous monitoring [414]. The limitations of current microfluidic devices for environmental monitoring have been critically assessed, and potential strategies to mitigate these limitations have been proposed [34]. For instance, the use of liquid reagents in colorimetric sensing necessitates the use of energy-demanding valves and pumps, limiting the lifetime of colorimetric systems in the field. Challenges exist in controlling the volume of samples exposed to stored reagents for a controlled sample–reagent interaction in polymer-based microfluidic devices [408]. The integration of different functional components in a single device and the precise control of the flow rate, pressure, and temperature of the fluidics is also a topic that requires further investigation [415].

### 5.3. Industrial Applications

Polymer-based microfluidic devices have applications in various industries, including chemical synthesis, food and beverage, and energy. Microfluidic devices are used for conducting chemical reactions with precise control over reaction conditions. Microfluidic devices, stemming from micro-electromechanical systems, have shown promise in building new chemical technologies with increased speed, reliability, and reduced sample consumption and cost. These devices enable precise, high-throughput, and automatic analysis of chemical synthesis processes, offering features such as high mixing efficiency, short reaction time, and controllable residence time [38]. Microfluidic devices provide precision control over reaction conditions by enabling continuous recirculation of droplets in a closed loop, maintaining low consumption of reagents and stabilized temperature, crucial for chemical reactions [416]. Microfluidic-based technology allows for the precise localization and controlled chemical treatment of structures on a surface, offering new opportunities for controlled assembly of structures and subsequent treatment [417]. Microfluidics also enables precise control of chemical reactions, yielding morphologically controllable particles with enhanced uniformity and explosive efficacy, particularly in the synthesis of energetic materials [418].

These devices enable the development of efficient and scalable processes for producing chemicals, pharmaceuticals, and materials. They act as reaction vessels for chemical and biological processes, reducing space, equipment costs, and reaction times while enhancing product quality [15]. Microfluidic devices play a significant role in drugs and nanomedicine production, diagnostic kits, and bioprocess design, allowing for rapid development of cell factories and bioprocesses [419,420,421]. They also enable the synthesis of advanced materials for various applications, including drug delivery, biological sciences, and tissue engineering, by providing highly controlled and rapid reactions [40]. The use of polymers, paper, and hydrogels, along with manufacturing techniques such as soft lithography and 3D printing, has made the development of low-cost microfluidic devices feasible, further enhancing their applicability in pharmaceutical and chemical industries [422].

Microfluidic devices can detect contaminants, monitor fermentation processes, and analyze the composition of food and drink products. Hence, they are suitable to be used for quality control and testing in the food and beverage industry. Microfluidic paper-based analytical devices (μPADs) have been proposed as effective tools for safety and quality monitoring in terms of microbial detection in food matrices [423]. Microfluidic technology allows for simple, rapid, and on-site testing, enabling timely, cost-effective, and accurate food safety control. These devices offer advantages such as reduced sample and reagent consumption, high sensitivity, automation, low cost, and portability, making them ideal for on-site detection, especially in low-resource areas [424]. The technology ensures reduced costs of analysis, enhanced mass and heat transfer, and improved analytical performances, making it a promising tool for faster analytical testing in the food industry [425].

Nonetheless, there is a need for standardization of materials, channel geometries, and process conditions to achieve maximum process efficiency in microfluidic devices [426]. The wide manufacturing of microfluidic devices demands intensive studies to be conducted for user-friendly and accurate food safety control, and the development of microfluidic devices for food safety monitoring requires addressing existing limitations and employing future trends in microfluidic design and fabrication processes [427].

Microfluidic devices are used in the energy sector for applications such as fuel cell development and oil analysis. They find a use in fuel cell development, offering benefits such as portability, faster mass transfer, high power density, and low cost [363]. Microfluidic devices also have the potential to improve oil recovery efficiency, reduce costs, and provide valuable insights into fluid behavior and reservoir characterization in the petroleum industry [428,429]. Microfluidic fuel cells can integrate flow of electrolytes, electrode–electrolyte interactions, and power generation in a microchannel, offering possibilities for rapid energy generation with high power density, low cost, and disposability [363]. They can be used to harvest energy from various sources such as glucose, microbes, and formic acid, without any metallic catalyst, for energizing low-power portable devices or applications [428,430]. Microfluidic paper-based fuel cells have been optimized to achieve maximum energy density, offering a cost-effective solution for energy harvesting applications [431].

Challenges such as bubble accumulation in microfluidic fuel cells have been addressed, emphasizing the importance of specific design elements for stable operation [432]. Future research paths include the evaluation of additive manufacturing for the quick prototyping and modification of three-dimensional structures replicating natural oil-bearing rock formations for improved understanding of oil recovery processes [433].

These devices can improve the efficiency of energy production and provide real-time monitoring of energy systems, as demonstrated using Raman spectroscopy to quantify chemical species in microfluidic devices [434]. The integration of microfluidic systems in biosensors allows for precise control of flow, better mixing of analytes and reagents, and enhanced sensitivity of detection, enabling real-time monitoring with high precision and accuracy [435]. Furthermore, microfluidic devices have been explored for energy storage systems, offering unique architectures and enhanced performances for storing various forms of energy such as electrochemical, biochemical, and solar energy [428,436].

**Figure 8:**
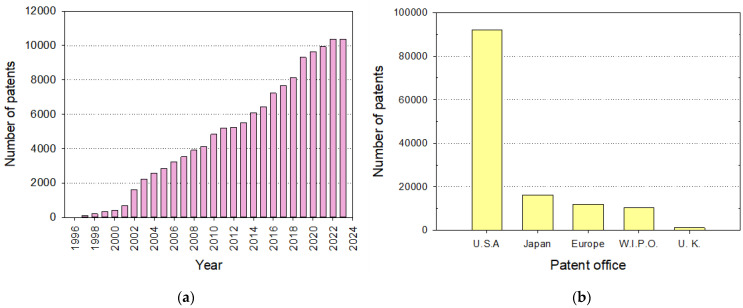
Number of patents in the field of microfluidics per year (**a**) and per patent office (**b**). W.I.P.O.: World Intellectual Property Organization.

The economic relevance of the industrial applications of microfluidic devices can be clearly appreciated in Figure 8a, which represents the number of patents that have been registered in this field. It is noteworthy that throughout the entire period 1997–2023, the yearly number of patents clearly exceeds that of published papers, which highlights the importance of this particular research area.

Figure 8b illustrates the noticeable inequality in the geographical origin of the patents, with the United States Patent & Trademark Office holding 92,265 patents out of the total 131,800 patents registered worldwide, vastly more than the Japan Patent Office (13,227).

### 5.4. Point-of-Care Diagnostics

Point-of-care (POC) diagnostics represent a significant application area for polymer-based microfluidic devices, enabling rapid and decentralized testing in various settings, from remote locations to emergency rooms [437]. These devices play a crucial role in the development of POC tools, offering rapid and easy-to-read diagnostic assays for various diseases and physiological conditions, which can be applied by non-medically trained persons. The use of microfluidic devices in POC diagnostics allows for the rapid and easy-to-read diagnostic assays of diseases such as COVID-19, allergies, cardiovascular diseases, tumors, and pregnancy, among others [438,439]. During the COVID-19 pandemic, microfluidic devices were developed for the rapid testing of SARS-CoV-2. These devices could process small samples and provide results within minutes, significantly enhancing testing capabilities [440]. On the other hand, continuous glucose monitors (CGMs) using microfluidic technology have revolutionized diabetes management, allowing for real-time monitoring and better glycemic control [441,442,443].

The integration of microfluidics and electronics has simplified the sample handling process, reduced sample usage, and lowered the cost of tests in POC diagnostics [444]. Microfluidic platforms provide an integrated, miniaturized, and cost-effective alternative to conventional POC devices, offering low sample volume and lesser time for detection [445]. The amalgamation of nanobiotechnology with microfluidics has given rise to highly selective and sensitive standalone devices that detect early disease onset and progression biomarkers.

POC diagnostic devices are designed to be easy to operate, offering rapid and robust testing at or near the point of care [446]. They aim to provide quick diagnostic information in non-laboratory environments, facilitating healthcare procedures and management [447]. The devices are intended to be portable, with advancements in technologies such as miniaturized transduction and lab-on-a-chip systems contributing to their portability and suitability for major infectious disease diagnosis [448,449,450]. Rapid operation and turnaround times are essential features of POC diagnostic devices, as they can significantly reduce the dissemination, morbidity, and mortality of infectious diseases [451]. Rapid diagnostics (<1 h) can contribute to controlling infectious diseases and antimicrobial resistant pathogens [452]. Polymer-based microfluidic devices meet all these criteria due to their low cost, flexibility, and ability to integrate multiple functions into a single platform.

While POC diagnostics offer numerous advantages, challenges such as sample handling, device calibration, equipment maintenance, calibration, and governance, and data interpretation remain. Innovations in materials and device design are addressing these issues, improving the accuracy and reliability of POC diagnostic tools [446]. However, advancements in biomedical engineering and information technology are expected to drive the creation of next-generation devices to meet current and emerging clinical needs [453].

## 6. Future Challenges and Developments

As polymer-based microfluidic devices continue to advance, several challenges must be addressed to fully unlock their potential across various applications, particularly in biomedical research. These challenges span material properties, device functionality, scalability, and ethical considerations. The following sections outline key areas where ongoing research and development efforts are focused.

### 6.1. Chemical Resistance and Gas Permeability

One of the major challenges is achieving adequate chemical resistance and low gas permeability. Polymers often exhibit poor resistance to certain chemicals and solvents, which can lead to device degradation and failure. Additionally, high gas permeability can result in the loss of volatile reagents and compromise the accuracy of assays. Ongoing research aims to develop new polymer formulations and surface treatments that enhance chemical resistance and reduce gas permeability. For example, the incorporation of barrier coatings and the development of cross-linked polymer networks can improve the chemical stability and reduce the gas permeability of microfluidic devices.

### 6.2. Advances in Materials Science

Advances in material science are addressing these challenges by developing new polymers and composites with enhanced properties. The development of fluorinated polymers and perfluorinated polymers offers superior chemical resistance and low gas permeability, making them suitable for demanding applications in chemical and biological analysis. Researchers are also exploring the use of hybrid materials that combine the advantages of different polymers to achieve the desired properties.

### 6.3. Integration of Multifunctional Systems

The integration of multiple functionalities into a single microfluidic device remains a complex task. Future developments will focus on creating multifunctional systems that combine fluid handling, sensing, and actuation in a seamless and compact design. This requires advancements in materials engineering, microfabrication techniques, and system integration. The development of hybrid materials and composite structures that combine the advantages of different polymers and other materials will be crucial for achieving multifunctionality. Additionally, novel fabrication techniques such as multi-material 3D printing and soft lithography will enable the creation of complex microfluidic systems with integrated functionalities.

### 6.4. Multilayer and Modular Microfluidic Devices

The development of multilayer microfluidic devices, where multiple functional layers are integrated into a single device, is an area of active research. These devices can incorporate fluidic channels, sensors, and actuators in a compact format, enabling sophisticated functionalities in a small footprint. Innovations in bonding techniques and interlayer connections are enhancing the performance and reliability of these devices. Researchers are also exploring the use of modular designs, where different functional modules can be combined to create customizable and reconfigurable microfluidic systems.

### 6.5. Ensuring Biocompatibility and Preventing Biofouling

Ensuring biocompatibility and preventing biofouling are critical for the successful application of microfluidic devices in biomedical research and clinical diagnostics. Biofouling, the unwanted adsorption of biomolecules and cells on device surfaces, can lead to device clogging and compromised assay performance. Advances in surface coatings and materials engineering are expected to improve the performance of microfluidic devices in biological environments. For example, the development of antifouling coatings and hydrophilic polymers can reduce biofouling and enhance biocompatibility. Additionally, the incorporation of bioactive materials and functionalized surfaces can improve the interaction of microfluidic devices with biological samples, ensuring accurate and reliable results.

### 6.6. Scaling up Production

Scaling up the production of polymer-based microfluidic devices while maintaining quality and performance is a significant challenge. Innovations in manufacturing processes, such as roll-to-roll printing, automated assembly, and high-throughput fabrication techniques, will be essential to meet the growing demand for these devices in various industries. The development of cost-effective and scalable manufacturing methods will enable the widespread adoption of microfluidic technologies in areas such as diagnostics, drug development, and environmental monitoring. Additionally, advancements in quality control and standardization will ensure the consistent performance and reliability of microfluidic devices.

### 6.7. Overcoming Regulatory and Standardization Challenges

The widespread adoption of polymer-based microfluidic devices is also contingent on overcoming regulatory and standardization challenges. Ensuring compliance with regulatory standards for medical devices, such as those set by the FDA and EMA, is crucial for the commercialization of these technologies. Additionally, establishing industry-wide standards for the fabrication, testing, and performance of microfluidic devices will facilitate their integration into various applications. Researchers and manufacturers are working closely with regulatory agencies to develop guidelines and protocols for the safe and effective use of microfluidic devices. This includes conducting comprehensive preclinical and clinical studies to demonstrate the safety, efficacy, and reliability of these devices. Establishing robust quality control measures and adhering to good manufacturing practices (GMP) are also essential for meeting regulatory requirements.

### 6.8. Ethical and Social Considerations

The development and deployment of polymer-based microfluidic devices raise important ethical and social considerations. Ensuring equitable access to these technologies, particularly in low-resource settings, is a critical challenge. Additionally, addressing concerns related to data privacy and security, especially in the context of wearable and implantable devices, is essential for gaining public trust and acceptance. Promoting the ethical development and use of microfluidic technologies involves engaging with diverse stakeholders, including patients, healthcare providers, policymakers, and the public. Researchers and manufacturers must prioritize transparency, inclusivity, and accountability in their work. Developing frameworks for ethical decision-making and fostering public dialogue on the benefits and risks of microfluidic technologies will help ensure their responsible and equitable use.

## 7. Conclusions

The synergy between polymer science and microfluidic technology has led to significant advancements in the field of microfluidics, driving innovations in materials and fabrication processes. Polymers, with their unique properties such as flexibility, biocompatibility, and structural integrity, have become indispensable in creating microfluidic devices with enhanced functionality and performance. Advancements in fabrication techniques, including replica molding, microcontact printing, solvent-assisted molding, injection molding, and 3D printing, have enabled the production of sophisticated microfluidic systems.

These innovations have expanded the application scope of microfluidic devices in various domains, including biomedical diagnostics, drug delivery, organ-on-chip models, environmental monitoring, and industrial processes. For instance, polymer-based microfluidic devices are now pivotal in developing point-of-care diagnostics, personalized medicine, and high-throughput screening platforms. Additionally, these devices are increasingly used in environmental monitoring for detecting pollutants and pathogens and in industrial processes for precise chemical synthesis and material production.

Despite these advancements, several challenges remain. Enhancing the chemical resistance and reducing the gas permeability of polymers are critical for ensuring the longevity and reliability of microfluidic devices. Many polymers still face limitations when exposed to harsh chemicals and solvents, which can degrade the device over time. Innovations in polymer chemistry, such as the development of cross-linked polymer networks and barrier coatings, are essential to addressing these issues.

Achieving multifunctionality in a compact design is another major challenge. Integrating various functions such as fluid handling, sensing, and actuation into a single microfluidic device requires sophisticated design and manufacturing techniques. Future developments may focus on multi-material 3D printing and hybrid fabrication methods to create more complex and integrated systems. Additionally, ensuring biocompatibility while preventing biofouling is essential for biomedical applications. Biofouling can significantly impair device performance; thus, the development of antifouling coatings and surface modifications will be crucial.

Scaling up production while maintaining quality and performance is another significant hurdle. Traditional manufacturing processes often face limitations in scalability and consistency. Innovations in high-throughput manufacturing techniques such as roll-to-roll printing, automated assembly, and continuous casting are necessary to meet the growing demand. Establishing robust quality control measures and adhering to stringent standards will ensure the consistent performance and reliability of these devices.

To address these challenges, future research could focus on developing advanced materials with tailored properties to enhance chemical resistance and reduce gas permeability further. For example, ongoing work on fluorinated polymers and hybrid composites could lead to breakthroughs in device durability. Additionally, leveraging machine learning and artificial intelligence for optimizing multi-material fabrication processes could significantly enhance the integration of multiple functions within a single microfluidic platform. New applications, such as personalized organ-on-chip systems for patient-specific drug testing, and innovations like self-healing microfluidic networks represent potential avenues for future exploration. Another promising direction involves the use of stimuli-responsive materials that can dynamically adjust their properties in response to environmental changes, enabling more adaptable and robust microfluidic systems. 

Future developments will also need to address regulatory and standardization challenges to facilitate widespread adoption. Compliance with regulatory standards for medical devices is crucial for commercialization. Establishing industry-wide standards for the fabrication, testing, and performance of microfluidic devices will streamline their integration into various applications. Additionally, ethical and social considerations, particularly related to equitable access and data privacy, must be addressed to ensure the responsible development and deployment of these technologies. Ensuring that these advanced technologies are accessible to low-resource settings and protecting user data will be critical for gaining public trust and acceptance.

The continued collaboration between material scientists, engineers, and biologists will be crucial for driving further innovations and realizing the full potential of polymer-based microfluidic devices. By overcoming the remaining technical, regulatory, and ethical challenges, the widespread adoption and impactful use of these transformative technologies in biotechnology, medicine, and beyond can be achieved. Future research and development efforts should focus on creating more robust, versatile, and scalable microfluidic systems to unlock new applications and improve existing ones. Through these collaborative and innovative efforts, polymer-based microfluidic devices will continue to revolutionize various fields, providing advanced solutions for complex scientific and industrial challenges.

## Data Availability

No new data were created or analyzed in this study. Data sharing is not applicable to this article.
